# Assessment of the quality of sexual and reproductive health services delivered to adolescents at Ujala clinics: A qualitative study in Rajasthan, India

**DOI:** 10.1371/journal.pone.0261757

**Published:** 2022-01-10

**Authors:** Radhika Dayal, Mukta Gundi

**Affiliations:** Department of Poverty Gender and Youth, Population Council, New Delhi, Delhi, India; University of Western Australia, AUSTRALIA

## Abstract

The Adolescent Friendly Health Clinic (AFHCs), a key component of the Government of India’s National Adolescent Health Programme a.k.a. Rashtriya Kishor Swasthya Karyakram (RKSK), aims to increase the accessibility and utilization of sexual-reproductive health services by adolescents and youth. However, low quality of care provided at AFHCs by counsellors calls for attention. We, thus, explore both the clients’ and providers’ perspectives using the World Health Organization’s (WHO) global standards for quality health-care services for adolescents to assess the quality of the sexual reproductive health service delivery at AFHCs in Rajasthan, India. We conducted a qualitative study, comprising observation of the service delivery using mystery clients (MCs) (n = 12) and in-depth interviews with the counsellors (n = 4) in four AFHCs. Interviews were transcribed in local language and were translated in English. The transcripts were coded thematically. Our study, using five of the eight WHO global standards for quality health-care services for adolescents highlighted several gaps in the quality-of-service delivery at AFHCs. We unearth various intricacies related to the quality of the services provided at the AFHCs by referring to the relevant input, process, and the output criteria of WHO global standards I, III, IV, V and VI. Our study calls for efforts to improve- (i) the counsellors’ competencies to increase adolescents’ health literacy on sensitive topics, (ii) the facilities at the clinic to ensure privacy, comfort and confidentiality of the adolescents seeking services, (iii) the referrals to improve appropriate package of services, and (iv) an overall environment to ensure an equity and non-discrimination for all the adolescents. Our findings unearth the barriers that both the service providers and the adolescents face at the AFHCs and underscore the need for regular monitoring and evaluation of the AFHCs to strengthen the facility-based intervention of the RKSK programme.

## Introduction

Adolescent Friendly Health Clinics (AFHCs) are one of the critical pillars of the National Adolescent Health Program in India- Rashtriya Kishor Swasthya Karyakram (RKSK) that seeks to enable all adolescents and youth to realize their full potential by making informed decisions concerning their health, and by accessing the services and support to implement their decisions [1]. AFHCs also known as the Ujala Clinics (UCs), were established in India to provide adolescent and youth-focused clinical and counselling services catering to their various health needs including those related to sexual and reproductive health (SRH); nutrition; violence etc. [2]. The RKSK program designed and implemented by the Ministry of Health and Family Welfare offered training of the Adolescent Friendly Health Clinics service providers such as medical officers, Auxiliary Nurse Midwives and Counsellors who provide these services at Adolescent Friendly Health Clinics in the government health system such as in Primary and Community Health Centers. The counsellors are trained on the technical details of various sensitive health topics to build their ability to educate, counsel or refer young clients to an advanced care as per the need. They are also trained to conduct the outreach activities for adolescents in educational institutes and in the community on health issues and make them aware of various available adolescent friendly health services. However, literature suggests some lacunae in training as the content related to sexual and reproductive health (SRH) information lacks focus and is inadequate in its ability to make the service providers comfortable in sharing the sensitive information [3]. A total of 7969 AFHCs have been established across India by 2019 and the number of dedicated adolescent health counsellors is 1671 [4].

The AFHCs, however, have not made significant inroads into providing services to adolescents. The awareness and utilization of AFHCs remains minimal [5–9]. Evaluation studies have reported a provision of inadequate and sub-standard services; a lack of privacy and confidentiality, and counsellors with an inadequate knowledge and experience as some of the barriers in accessing services at the AFHCs [3, 10]. Although evidence from India has brought forth how competing responsibilities of service providers and time constraints faced by young clients, together, affect the quality of services at AFHC [3], a comprehensive picture regarding the constraints faced by the service providers, and by the counsellors remains under-reported. The barriers to utilization of services at AFHCs include- low awareness among adolescents; socio-cultural misbeliefs that AFHCs are ‘only for the sick and for the girls’; limited mobility among girls; preference to consult a friend or family over a counsellor; a lack of money and family support to seek counselling and services; a lack of supportive supervision to health-care providers, and a poor quality of care [3, 6].

These lacunae call for an urgent need to develop and evaluate interventions that focus on strengthening and improving the quality of the services provided at the AFHCs. In the past, few evaluations in India have used participatory research methodologies to assess the quality of services which have proven to be reliable, valid, feasible and acceptable [11, 12].

A systematic review of 30 evaluation studies in India highlighted that the definitions of ‘quality of care’ in the reference of AFHCs were inconsistent [13]. A study conducted in Maharashtra, India describes the feasibility and usefulness of using the WHO framework to monitor and improve the quality of services [14]. A similar study conducted in Tanzania that uses this framework describes the challenges that the Ministry of Health and Social Welfare, Tanzania, identified and the way in which those challenges were addressed with the support of WHO, UNFPA and other partners. This study indicated that the development and implementation of quality standards was a useful means of ensuring efforts to make health services adolescent-friendly [15].

Furthermore, evidence from India is primarily based on the quality assessment at the health facility level but lacks the global standards approach to assess different aspects of the quality of adolescent health services provided at these clinics. Such an approach could provide a benchmark for the development, implementation and the standardization of the quality improvement initiatives and adolescent service provision across India. This approach can not only reduce the variability in quality of services but could also benefit in comparing the evaluation findings across the various states in the developing countries to suggest the potential benefits of disseminating the validated tools for shared use [13, 16].

Therefore, there exists a need to identify approaches to enhance service delivery through AFHCs and to generate the demand for such services among adolescents and youth. There is also a need to identify what services adolescents and youth require, what is feasible at community and facility levels, and what is feasible for implementation by different cadres of health care providers [17]. Hence, it is essential to explore the quality of healthcare services provided at Ujala clinics (UCs) adhering to the WHO’s global standards for quality health-care services for adolescents [18].

To address this knowledge-gap, the Population Council assessed services provided at the AFHCs also known as the Ujala clinics (UCs) in Dholpur and Karauli districts in Rajasthan as part of the UDAAN evaluation baseline. The UDAAN intervention, which is currently being implemented in Rajasthan, aims to reduce adolescent fertility by focusing on three strategies: 1) strengthening the scholarship mechanisms to increase enrolment and retention in secondary schools; 2) improving knowledge, attitude, and practice (KAP) with regards to sexual and reproductive health (SRH) among adolescents, using a human-centred design approach, and 3) expanding the contraceptive use and method mix for young women. One of the objectives of the evaluation is to assess the quality of SRH services provided by the counsellors at UCs and the counsellors’ perspectives on SRH issues and the challenges faced by them in providing the counselling services to adolescents.

To the best of our knowledge, this is the first qualitative study in India that has used the WHO’s global standards of quality health-care services for adolescents [18] to analyse the data for the quality of services provided at UCs based on both the clients’ and providers’ perspective in Rajasthan, India.

## Methods

### Ethics statement

The study protocol was approved by the Institutional Review Board of the Population Council. We followed strict measures to ensure that research ethics were followed throughout this study as it covered several sensitive topics on SRH of adolescents. As the study sought to understand the quality of services received by adolescents using mystery clients (MCs), we recruited adolescents and young men/women aged 18–22 as mystery clients (MCs) from the villages not in the proximity to the clinics to ensure anonymity. Precautions to maintain confidentiality were taken to avoid the risk of teasing, harassment, and harm to MC’s reputation. Based on our previous experiences of working with adolescents on various sensitive topics, we prepared the interview guides that were age-appropriate and culturally relevant. MCs were provided training for two days in ethical issues. Informed written consent was sought from everyone to be interviewed. The interviewer was also required to sign a statement that she or he had explained the content of the consent form to the respondent. Additionally, the study team had approached the government officials at the district level, and public health authorities to explain the details of the study and had sought permission from them to conduct this study in the selected UCs.

### Study setting

The study took place in Dholpur (intervention district) and Karauli (matched comparison district) in Rajasthan state in India. To observe the services provided by the counsellors at AFHCs, also known as UCs, MC visits were conducted in four UCs–two each in the intervention and the comparison block purposively selected for the current study. In-depth interviews (IDIs) with the counsellors were conducted from the same blocks.

### Study design

We followed a qualitative approach to gain an in-depth knowledge of the quality of services provided at the UCs from both the client and providers perspective by developing study instruments (six scripted scenarios; debriefing guide, and IDI guide). To capture the client’s perspective, we followed the procedure for conducting MC visits as suggested in the guide published by Pathfinder International [19]. Immediately after the visits, MCs debriefed the study team at a convenient place. The effectiveness of using this approach can be substantiated by a recent systematic review of 30 studies published across low- and middle-income countries that used the MC methodology as part of their research to understand adolescents’ access to SRH services [11].

To explore the providers perspective we conducted the IDIs of total four counsellors (three men and one woman from the intervention and the comparison arm) to better understand their role in the clinics and the constraints they faced in providing the services to clients. We thematically analysed the qualitative data [19].

### Participant recruitment

With the help of the local NGOs and an embedded evaluator based in the intervention district, we identified the MCs and the counsellors using purposive sampling method. Six women and six men aged 18 to 22 were identified. One of the reasons for selecting MCs in this age range was to ensure they were not school going to maintain their anonymity. Additionally, as the methodology required the MCs to perform the script in a trained manner, we wanted to engage older adolescents and young adults who could understand the intricacies of performing as per their scripts. They were residents of a village that was not in the proximity to the clinic; demonstrated talent for roleplaying and displayed openness about discussing SRH matters. We ensured that MCs were not identifiable by community members, health-facility staff or by the counsellors.

### Training of MCs

We trained the MCs to familiarize them with the likely scenarios at the clinic. We provided guidance in phrasing their questions to the counsellors, conducted role-plays for all the possible scenarios and briefed them regarding the potential criteria for evaluating the quality of services. The scenarios assigned to the MCs included: seeking 1) treatment for menstrual problems, 2) advice for resisting boyfriend’s coercion to engage in sex, 3) information about how to avoid an unwanted pregnancy, 4) information on the use of condom, 5) information on masturbation, and nocturnal emissions, and 6) treatment for sexually transmitted infection. The scripted scenarios and the debrief checklist for the MCs are summarized in ‘S1 Appendix’.

MCs were informed about each clinic that they needed to visit and were asked to observe everything that the counsellor did or said without mentioning any affiliation with the study during their visit. They were requested to refuse to undergo any physical examination. To keep the experience of MCs as close as possible to that of the genuine clients, their visit was restricted to just one clinic per district. MCs made visits in pairs—one was assigned to serve as the client and the other to serve as an observer—to minimise the recall bias [19]. Each MC and the observer were paid a monetary compensation of Rs. 6800 (USD 95) for participating in a two-day training. Overall, twenty-four MC visits—six visits per clinic—covering all six scenarios were successfully completed. The counsellors to be interviewed were selected from the same clinics where MC visits were made.

### Data collection

Semi-structured questionnaires were translated into the local language (Hindi) by a professional translator proficient in translating the study tools from English to Hindi. The questionnaires were used for a separate debriefing of each MC and the counsellor. They were probed about (i) their experience in availing the services at the clinic (e.g., the registration process, waiting time to see the counsellor etc); (ii) the information given by the counsellor (e.g., content, comprehensiveness, counselling skills), and (iii) the counsellor’s behaviour toward the client (e.g., objectivity, level of comfort in discussing SRH matters with the client).

The study-team conducted the IDIs of the counsellors to understand- (i) their role as a counsellor; (ii) the details of the training they received; (iii) their perceptions about the training, and service utilization, and (iv) the constraints faced by them while providing the services to adolescents and youth in general and through UCs.

All interviews, carried out in Hindi, were audio-recorded, and key points were noted. They were transcribed verbatim and then translated into English. The MC and observer debriefing interviews lasted on an average between 30–60 minutes and the IDIs with the counsellors lasted for about 30–45 minutes.

### Data analysis

Grounded theory approach was used to analyze the data thematically. Our analysis involved five key stages: familiarization; identifying a thematic framework; indexing; charting, mapping, and interpretation [20, 21]. In the first stage, RD and SS read the interview transcripts to get familiarized with the data. In the second stage, RD and SS developed a coding framework based on the topics used to guide the debriefing interviews conducted with the MCs and the IDIs with the counsellors. The new themes that emerged from the data were re-organized in the coding framework that was developed based on the descriptive themes by RD and SS, independently, followed by consensus. In stage three RD and SS, coded the entire data using the framework established in stage two. In stage four, RD and SS undertook a process of mapping and interpretation i.e., established charts were used to explore the range and the nature of phenomena. Any emerging associations between sub-themes were identified to explain the findings a process of mapping and interpretation was undertaken by RD and MG. Our finalized subthemes fitted with five out of the eight WHO global standards for quality health-care services for adolescents in terms of- a characteristic of the service that needs to be in place (input criterion) or implemented (process criterion) in order to achieve the defined standard (output criterion). Final sub-themes derived from the analysis explored the relevant criteria from five out of the eight global standards of quality health-care services in terms of- a characteristic of the service that needs to be in place (input criterion) or implemented (process criterion) to achieve the defined standard (output criterion). The sub-themes derived from our analysis explored the criteria from five global standards viz.- adolescent health literacy (Standard-1), appropriate package of services (Standard-3), providers’ competencies (standard-4), facility characteristics (Standard-5) and Equity and non-discrimination (standard-6) [18]. Some criteria from these standards as well as the remaining three standards viz. community support (Standard 2), data and quality improvement (Standard 7) and adolescents’ participation (Standard 8) were not explored due to the limitations posed by the methodology as MCs made single visit to each UC. Additionally, our methodology included adolescents who were trained as MCs, which limited our understanding of the criteria measuring the improved awareness among adolescents on various health topics. The limitations of our methodological approach did not allow us to understand whether (i) the system ensured to follow processes to gain community support; (ii) the staff at the UCs had support to collect, analyze data for quality improvement, and (iii) whether adolescents were actively involved in planning, monitoring, and evaluating the services [18]. It is to be noted that the scripts of the MCs were focused on SRH among adolescents, hence, did not cover other health-related topics such as- nutrition. We summarize the intricacies related to the quality of the health-care services provided at four UCs by referring to the relevant input, process, and the output criteria of WHO global standards I, III, IV, V and VI. in ‘S2 Appendix’.

## Results

The average age of the female MCs was 21 years (SD 1.09) of male MCs was 22.1 years (SD 0.75). About 11 MCs (six females and five males) had completed their graduation and one male had completed his higher-secondary education. The average age of the male counsellors was 31.3 years and female counsellor was 28 years old. All the counsellors had completed their post-graduation in social work or sociology. Our results are based on IDIs with one counsellor in each UC [n = 4] conducted by the research team member. Additionally, a total of 12 MCs [6 male and 6 female] made 24 visits to these four UCs [6 visits per UC] after they were trained for the scripted scenarios. Table 1 summarizes the socio-demographic profile of the MCs, counsellors, and the description of the study components. Table 1 summarizes the socio-demographic profile of the MCs, counsellors, and the description of the study components.

**Table 1:** Summary of the sociodemographic profile of the study participants and study components.

Study components	Respondents	Sample size targeted	Sample size reached
MC interviews	18 to 22 years young men and women residing in a village not in the proximity to the clinic and willing to act as MCs	12 (6 males and 6 females) 24 visits to UCs; 6 visits per clinic and two clinics each in intervention and comparison arms; therefore, 12 each in intervention and comparison arms at baseline.	12 (6 males and 6 females) 24 visits to UCs; 6 visits per clinic and two clinics each in intervention and comparison arms; therefore, 12 each in intervention and comparison arms at baseline.
In-depth interviews with counsellors	Counsellors serving at the selected UCs	8 counsellors, 4 each in intervention and comparison arms	4 counsellors, 2 each in intervention and comparison arms

### 1. Adolescent health literacy [WHO global Standard I]

#### 1.1 Directional signs

Only one of the four clinics had directional signs both outside and inside the clinic displaying the information and the direction for UC with RKSK logo and a slogan written, ‘*Swasthya Suljhe Kishor Badhe Pragati ki Ore*’ (healthy teens lead to progress). A Majority (19/24) of the clients sought help from the hospital staff to locate the clinic. Most of the staff from all the four clinics were aware of the clinic’s location and were friendly in directing the clients to UCs. Only in one case, a physician was found unaware of the location of the clinic. In one fifth of the cases (4/19), clinic staff asked the reason of the visit and personal details to the clients, indicating their lack of sensitization in maintaining client’s confidentiality.

An 18-year-old unmarried girl seeking information on unwanted pregnancy stated, “I *asked the compounder about the Ujala clinic and the counsellor*. *He started asking me my village name and other details*. *Then he pointed towards another boy sitting there and said that he is from the same village and you might know him*.*”*

#### 1.2. Information, Education and Communication (IEC) materials for adolescents

The clients reported that only a limited IEC material on SRH was displayed in the waiting area of all the four UCs. The information on menstrual hygiene with pictorial images and easy-to-remember messages was displayed which most MCs found useful. Only one client came across a poster informing the transmission of HIV through an infected syringe. The same clinic reportedly had a calendar on the counsellor’s desk that displayed the information on condoms.

A 17-year-old unmarried boy seeking information on STIs stated that “*this information is useful for adolescents as they don’t know the benefits of using condoms and how it can prevent unwanted pregnancy and infections*.” Two out of the four clinics had posters displaying the information on family planning methods such as injectables and contraceptive pills.

“*The poster on oral contraceptive tablets was informative as it described in detail about the timing and the frequency of taking this tablet*. *The poster for the injectable contraceptive had information on when to take it*, *ease of using the injection*, *benefits of using it like preventing pregnancy for the next 6 months for one-time use*. *It also mentioned that there will be no side effects*, *and it’s not inglorious to take the injection”*

*-* MC, 17-year-old, unmarried boy requesting condoms.

#### 1.3. Health education and counselling services provided by the counsellors

Across all the visits, although counsellors seemed to provide the basic health education to MCs, except one counsellor, others were found to provide some misinformation while counselling on the sensitive topics such as masturbation. We also received varied responses on the educational tools and materials used by the counsellors to counsel on these topics. During our interviews with the counsellors, three mentioned about referring to informative posters that were displayed in the clinic, in addition to referring to books or comics and distributed these materials to adolescents. On the contrary, none of the counsellors mentioned ever distributing any flyers, leaflets, or brochures to adolescents after the counselling sessions.

A male counsellor revealed, “*we don’t have any pamphlets or brochures to give the clients them*. *We provide them with our contact number and the toll- free number of 104/108 or only suggest them about the Ujala Clinic*.*”*

The narratives of some of the counsellors indicated that the counsellors were provided the support materials during their training which would help them in offering the package of services and counselling to adolescents. Our debriefing sessions with MCs indicated that the counsellors neither physically referred to any support materials nor handed it out to them while providing the counselling services. All the counsellors mentioned using soft skills (maintaining an eye-contact, keeping hands in an open position to build a rapport; assuring privacy; listening patiently) while providing the information and services to their clients.

#### 1.4. Reproductive health and hygiene

In two scenarios (unmarried girl experiencing menstrual problems; unmarried boy enquiring about masturbation and nocturnal emission), the counsellors reportedly shared an information highlighting the importance of reproductive hygiene. Male MCs were advised regarding genital hygiene while female MCs were advised merely regarding menstrual hygiene. Majority of female MCs were advised to use sanitary napkins instead of cloth-pads; to change the napkins regularly; to adopt an environment-friendly disposal of the napkins; and to wash and dry their cloth-pads in the sun, in case of use. A 17-year-old male MC stated that few counsellors advised male MCs to maintain genital hygiene by washing their genitals on a daily basis; washing and drying undergarments regularly, and by using cotton undergarments instead of the synthetic ones. A 17-year-old unmarried boy enquiring about nocturnal emissions recalled that the counsellor advised him to “*pull back the foreskin and clean it every 5–10 days with water*.*”*

Although MCs perceived the interaction on the reproductive health and hygiene matters with the counsellors to be positive; it was often found to have misinformation.

#### 1.5. Contraception

Condoms were the most advised contraceptive method by the counsellors to most of the MCs (both males and females). However, the details shared regarding the usage and the accessibility of the condom use varied. The male counsellors demonstrated confidence in discussing and demonstrating how to use a condom, whereas the female counsellor suggested the clients to read the instruction on the condom packet. Counsellors though discussed the advantages of the condom in detail, they reportedly refrained from describing any disadvantages with the MCs.

A 17-year-old, observer unmarried girl resisting boyfriend’s coercion to engage in sex stated that *“I asked him for the benefits of a condom*. *He said there are three benefits*. *Firstly*, *it prevents pregnancy; secondly it prevents transmission of diseases and finally*, *if by chance the girl gets pregnant and the family forces her for a medical examination*, *the condom prevents the hymen from breaking and maintains the privacy of the person*.*”*

The other contraceptive methods discussed mostly with the female MCs were copper-T, emergency contraceptive pill, female condom, injectable contraceptives, and oral contraceptive pills. None of the counsellors provided any information on the availability, accessibility, merits, and the demerits of these methods. Furthermore, in some instances, the counsellors advised against the use of Copper-T, as reflected in the quote below.

A 17-year-old, MC, an unmarried girl pressurized by her boyfriend to have a sexual intercourse stated *that “the counsellor informed me about Copper-T but said it is unnecessary to use as it is mainly used by ‘married couples’ to have age gap between children*.*”*

#### 1.6. Unwanted pregnancy

The female MCs seeking services related to unwanted pregnancies found that although two counsellors mentioned about the abortion pills and safe surgical abortion practices none of the counsellors provided detailed information and adequate guidance on legal abortion services, Medical Termination of Pregnancy Act, risks of and complications in unsafe abortion.

An 18-year-old MC (an unmarried girl) seeking information on unwanted pregnancy stated that “*He said that rather than doing it at home through a tablet it is better to get it (abort) in the hospital so that no one at home knows*, *since in the hospital it will be cleaner’*.

Two counsellors advised the female MCs to adopt effective communication, negotiation, and refusal skills to counter an unwanted sexual pressure and to employ the safer-sex strategies.

#### 1.7. STIs and HIV/AIDS

Our data from MC debriefing sessions highlighted that some of the counsellors provided an incorrect information on the causes, symptoms, and the treatment of STIs. One counsellor explained that STIs could be caused *‘if one had sexual relations with someone in an unhygienic place; indulges in excess sex and had an intercourse during a girl’s menstruation’*. Whereas information on HIV entailed informing MCs about unprotected sex causing HIV infection, explaining them about the stigma attached to people living with HIV, and mentioning about mother to child transmission during pregnancy, childbirth, and breastfeeding.

#### 1.8. Outreach activities to promote health and increase adolescents use of services

All the counsellors reported to have a monthly plan to conduct outreach activities; however, they were able to share limited details on activities conducted in the schools and nearby communities. The counsellors are often accompanied by the frontline healthcare providers i.e., the Anganwadi workers (AWW) and ASHAs [Accredited Social Health Activists] or. The primary role of AWWs under the Integrated Child Development Scheme (ICDS) is to support the supplementary nutrition and child development programmes and activities to combat the issue of child undernutrition [22]. The role of ASHAs is to be a village-level health activist to create health awareness and mobilize the villagers for demand, utilization, and accountability of the local health services. ASHAs also provide minimum primary health care and whenever necessary, make referrals of the patients to an appropriate health care facility [23]. The counsellors, along with ASHAs and AWWs mobilize the adolescents from the communities to conduct the outreach activities to increase their awareness about UCs and the services available at the centers.

Two counsellors discussed the constraints faced in conducting the sessions in schools due to a lack of support or interest from the school authorities. A male counsellor stated that *“many schools don’t have prior knowledge about counsellors making a visit and conducting sessions*. *Schools often question the creditability of our work*. *Schools are not mandated by the government to allow the counsellor to take sessions”*.

To generate an awareness and interest among adolescents and parents regarding health issues faced by adolescents, and to improve parent-child communication on SRH, the counsellors reportedly conducted puppet shows, health camps and games. A male counsellor explained that “w*e conduct outreach programs or organize health camps where they undergo a screening process*, *and we provide them with required medication*. *Apart from this we conduct activities like games*. *With this their hesitation reduces and there is an increase in adolescent’s motivation and confidence levels*.*”*

Two counsellors mentioned about Adolescent Health Days which are conducted in schools or Anganwadi centres every quarter. While talking about this day, a male counsellor in the comparison arm stated that

“…we measure their height and weight to check for those who are anaemic, we provide them information about counselling services available at Ujala clinics, helpline number (104/108) for more information and the importance of having nutritious food.”

### 2. Appropriate package of services provided to MCs [WHO global standard 3]

#### 2.1. Referral linkages

All counsellors reported referring the cases of substance abuse, alcohol addiction, STIs, menstrual pain, unwanted pregnancy, and nocturnal emissions to a more qualified healthcare provider. For instance, a male counsellor in the comparison arm stated that *‘if we get a case of an unwanted pregnancy and the girl wants to abort the child*, *I will refer her to the doctor*, *however*, *if the girl wants to keep the child*, *then I will guide the girl with the help of ANM or ASHA on various aspects of pregnancy*, *how to take care of the child and so on*. *In any case*, *we follow up with the ANMs on the client’s pregnancy*, *health and what reports look like*.” Another counsellor mentioned to have consulted the District Programme Manager in case of any legal complexity.

Our interviews with MCs with scripts depicting nocturnal emissions, STIs and teenage pregnancy, however, indicated discrepancy in referral linkages as only a few of those with symptoms were advised by the counsellors to take doctor’s advice.

### 3. Providers’ competencies [WHO global standard 4]

#### 3.1. Selection process and training received

All the counsellors reported that they were selected through a formal, rigorous application and interview process conducted by the National Health Mission in 2016. All of them were able to specifically describe their role.

Their training was reportedly conducted by the health departments, NGOs, academic institutions, and private development organizations that helped them learn about the RKSK programme, ways to strengthen the counselling skills, and the details of the UDAAN programme. Two of the four counsellors mentioned to have received the training on POCSO (Protection of children from sexual offense act), the Pre-conception and Pre-natal Diagnostic Technique Act.

#### 3.2. Counsellors’ competencies

The narratives highlighted that all the counsellors had limited knowledge and competencies in providing accurate information on the sensitive topics. Several counsellors mentioned that the bodily changes were “*same across all the adolescents*.” Additionally, many counsellors advised the female MCs for inculcating ‘healthy lifestyle’ to regulate their menstrual cycle without suggesting receiving any medical help for her menstrual ailments.

A 17-year-old unmarried girl experiencing menstrual problems stated that “*the counsellor mentioned that it is normal to experience pain during menstruation*, *and I should continue to do my daily tasks*. *The counsellor also advised me to consume green vegetables*, *hot milk and not take spicy food from outside to regulate my menstrual cycle*.*”*

None of the counsellors were found to explain the stages of menstruation cycle, the details, and the management of menstrual disorders to their female clients. An incomplete knowledge shared by the counsellors on the menstrual cycle, the reasons for menstrual pain and the most fertile time for a girl is visible from the following quote-

*"He told us that during the menstrual cycle*, *blood becomes thicker and clotted because of which females experience pain*. *It happens because the lining inside the uterus breaks and causes pain; this is a natural process"*, A 17-year-old MC stated. The causes of frequent masturbation were also found to be unknown among many counsellors. Two counsellors mentioned ‘it could cause weakness, mental distraction and addiction’. All counsellors were found to mention that masturbation and nocturnal emission was a “natural process and it happened due to the changes in the hormones.” However, two out of the four counsellors believed it was “wrong thing to indulge in it.” As the following quote suggests-

“I tried to make him understand that this was a bad habit, and he should try to quit it. I told him to replace it with any other activity.”

- A male counsellor from comparison arm

The counsellors did not seem to be fully aware of whether girls experience nocturnal emissions. A 17-year-old MC said that ‘I asked whether girls also experience the similar problem of wetting their pants [*sic*], to which the counsellor replied by saying that they do not experience it to such a great extent as men would.’ He said that if guys experience it twice or thrice a month, girls may experience it once a month”.

The findings highlighted that the counsellors had limited information about addressing the misconceptions and myths that the clients possessed. In only one of the eight visits, the counsellor was found to clear the client’s misconception regarding masturbation as reflected in the below quote-

“*the counsellor cleared the misconception by giving an analogy of a jug filled with water to explain how at this age*, *due to hormonal changes when the sperm count increases*, *it causes the body to release them & one of the ways to do so is by masturbation*”.

- A 16-year-old, MC, unmarried boy

None of the counsellors were found to share the accurate information on safe and confidential testing for STIs or HIV. In three out of the eight MC visits, the counsellors mentioned a need for a STI test “*only in case of serious symptoms such as blisters in private parts*.”

Our data revealed that all the four counsellors were aware of the importance of maintaining privacy and confidentiality while providing SRH services and of building a rapport to make the adolescents comfortable for discussing their health issues. A male counsellor stated that “*it is mandatory to inform the client that privacy and confidentiality of information will be maintained*, *and no information or details will be shared with anyone else*.” The narratives of 18 out of 24 MCs indicated that the counsellors assured confidentiality during the session. An 18-year-old, MC, unmarried boy with a scripted scenario enquiring about masturbation and nocturnal emissions stated that “*the moment we entered; the counsellor made us close the door*. *He assured us that the matter discussed would not be disclosed to anyone else*. *He did not ask me about my parents name and my phone number*.*”*

However, the clients in many visits reported a breach in their privacy and confidentiality as MCs reported disturbance from the staff and other patients visiting the clinic during the counselling sessions. In 4 out of 24 visits, the MCs reported that someone was present during the session. A 17-year-old MC, unmarried girl experiencing menstrual problems stated that “t*he person who accompanied us to the Ujala clinic was present there the whole time till we left*. *The door was open and anyone standing close to the door could have heard the conversation*. *Another person came inside the room to tell the doctor that he had a phone call*.*”*

In as frequent as half of the visits (12/24), the clients reported of people coming in between the counselling sessions. This was mainly due to the location of the clinic which led to disturbance and visibility by other outside the clinic. A 17-year-old MC, an unmarried adolescent boy requesting condom stated that *“the counsellor did not close the door*, *even before we could start the conversation*, *two clients came in and disrupted our conversation and asked the counsellor that he has been experiencing nocturnal emissions and if that could be a problem*.*”* However, during the IDI with the same counsellor, she mentioned that “*we assure the client that confidentiality of information will be maintained and ensure that no one else is in the room during the counselling session*.*”* Only one counsellor mentioned about the need to take parental consent before opting for surgery.

#### 3.3. Supportive supervision to improve providers performance

Although three counsellors mentioned about the supervision support for their work, only one counsellor shared the detailed information on supervision activities conducted by the officials to ensure smooth implementation of the program activities. Furthermore, the counsellor reported of monthly visits conducted by the chief medical officer, where the counsellor needed to submit the programme data and report; and also got an opportunity to share the challenges and new experiences faced during providing the services.

### 4. Facility characteristics [WHO global standard 5]

#### 4.1. Registration process

In only two out of 24 visits, the client had to go through the registration process, when the counsellor advised to consult the doctor. In both cases, the male MC had visited the counsellor to seek the information on STIs when he had to pay a registration fee of INR five (USD 0.07) and were handed over the prescription, while, in one case, the name, age and place of residence was inquired from the MC.

#### 4.2. Waiting time

Some counsellors made efforts to be punctual to work despite the odds faced due to long distances; a lack of transportation and managing family-work balance. A male counsellor from the intervention arm said, “*my work as a counsellor is very satisfying and make me very happy to provide knowledge to adolescents on SRH issues that are not much talked about in our communities*. *It gives me satisfaction that I am able to give back to my community*.*”* Furthermore, most of the counsellors were found spending on an average 30–35 minutes with each client. Differences in waiting time to consult the counsellor did not differ much, by the sex or the script of the client.

#### 4.3. Description of UC (waiting area outside the clinic)

All MCs reported to have waited in the area outside the clinic with a seating arrangement. Three out of four clinics had a separate room for the UC. In one health facility, the clinic was inside a doctor’s consultation room with a common entrance, but a cardboard used as a partition. Our data revealed mixed findings regarding the infrastructure of the facility. For instance, 17 out of the 24 clients found the clinic to be ‘*overall clean from inside and outside’*; 16 of the 24 clients found drinking water to be available at the facility, and six out of the 17 MCs who visited the toilet mentioned it to be clean.

#### 4.4. Supplies and medicines

Some female clients (4/24) reported to receive a packet of sanitary napkins, in addition to a pain-relieving tablet and iron tablets. In only few instances (5/24), clients reported that they were provided condoms. Information regarding how to use the pregnancy kit was received by some clients (5/8) and while few (3/8) were offered the pregnancy kits. A 17-year-old, MC, an unmarried girl resisting boyfriend’s coercion to engage in sex stated that, *“The counsellor drew it on a newspaper and told us that one needs to put two drops of urine sample in the space provided and if the test shows one pink line it means the women is not pregnant*, *however*, *if it shows two lines it means that she is pregnant*.*”*

She further added that *“The counsellor offered me a pregnancy kit*, *but I refused by saying that I will buy it from the market*. *He told us that he will give it for free in the clinic and the market price is 50 rupees*.*”*

### 5. Equity and non-discrimination: [WHO global standard 6]

One-third of the clients reported some counsellors’ behaviour was “*judgemental and biased*” as the counsellor had judgemental attitude towards unmarried adolescent girls engaging in premarital sexual activity. A 17-year-old, MC, an unmarried girl seeking information on unwanted pregnancy mentioned that *“the counsellor said that I should have not done this (have had sexual inercourse)*, *as it is wrong to do before marriage and will ruin my family’s reputation*. *If my family will get to know hey might even kill me*.*”* Additionally, a 19-year-old, *MC*, an unmarried boy requesting condoms said that *“the counsellor remarked that both of us couldn’t marry (inter-caste)*, *and that the marriage will be decided by the family*, *not by us*.*”* However, the same counsellor, when counselling on masturbation and nocturnal emissions, was found by the MC to be “*friendly and non-judgemental*.*”*

All clients reported that one female counsellor would rush while providing the counselling services. The clients saw her busy on the phone, found her to be disinterested, distracted and worked up in the clinic. A 18-year-old, *MC*, an unmarried girl seeking information on an unwanted pregnancy stated that *“the counsellor had got her baby to the clinic and was busy taking care of her*. *She seemed to be in a hurry to go and wanted to end the converstion*.*”* Such instances reflected not only a lack of training on counselling skills but also the challenges of balancing work and family life. The same female counsellors reflected that “*it becomes a tough condition and gets difficult for me to manage work and family*. *I have two kids and for one child who goes to school*, *I have to get him ready*, *pack his lunch and bag*.*”*

All counsellors either seemed uncomfortable or shy in providing the counselling services to the unmarried clients of the opposite sex. For instance, an 18-year-old unmarried girl seeking information on unwanted pregnancy mentioned *“the male counsellor asked me to bring my partner to the hospital from where he can get the condoms*.*”* Similarly, the counsellors also expressed a need to have both, a male and a female counsellor available at the clinic.

Clients who perceived themselves to have been respected, cared by the counsellor and felt unjudged were satisfied with the quality of the services provided.

## Discussion

This study, conducted in one of the poorest states in India using the mystery client methodological approach, highlighted some key gaps in the adolescent healthcare service such as issues with privacy and limited knowledge on some key SRH topics among the counsellors. To our knowledge, this is the first qualitative study capturing the quality of services provided at UCs from both, the clients’ and the counsellors’ perspective in Rajasthan, India using the WHO’s Global standards of adolescent health-care services framework that brought forth how social norms and gender bias coupled with existing lacunae in the service could impact adolescents’ healthcare ability and experiences.

The WHO framework enabled us to examine the quality health-care service for adolescents in the UCs in Rajasthan. Specifically, our study brought forth the gaps in the availability and the effective use of the IEC materials and tools; referral linkages; maintenance of the privacy and confidentiality of the clients in addition to the non-supportive socio-cultural norms and attitudes related to adolescents’ SRH among community-members. These findings corroborate the findings from previous studies which have emphasized the need to improve the quality of AFHS services, increase knowledge on SRH issues among clients and providers; and provide gender sensitive training to the counsellors, especially on maintaining privacy and confidentiality of information [11, 12]. Additionally, by adopting a standardized approach to measure the quality of services provided from both the clients’ and the counsellors’ perspective, we were able to unearth various factors and processes contributing to these lacunae. Our study design helped in drawing inferences on the effectiveness of the intervention from both the intervention and comparison arms and provided a comprehensive picture of which strategies need further strengthening.

The MC methodological approach helped us unearth the nuances of the experiences and challenges in SRH service provision by the counsellors and their behaviours towards the MCs. It also helped to gain insights from adolescents’ perspective on the quality of the SRH services they receive at the UCs and how it can potentially affect their attitudes towards SRH furthering the impact on SRH outcomes.

The present study has utilized five WHO standards to assess the quality of SRH provided by counsellors. The standards act as benchmarks against which quality of health care provided to adolescents could be compared. The WHO framework helped in identifying the good practices and services provided by the counsellors and the gaps identified that may require support in improving the services they offer [24]. A study conducted in Maharashtra, India describes the feasibility and usefulness of using the WHO framework to monitor and improve the quality of services [14]. A similar study conducted in Tanzania that uses this framework describes the challenges that the Ministry of Health and Social Welfare (MOHSW) Tanzania identified and the way in which those challenges were addressed with the support of WHO, UNFPA and other partners. This study indicated that the development and implementation of quality standards was a useful means of ensuring efforts to make health services adolescent-friendly [15].

Our study findings highlight various gaps in the SRH healthcare service delivery at UCs in Rajasthan using the selected criteria for five WHO global standards. We found that the health facility infrastructure presented varying levels of barriers which are easily addressable (such as signage, display of IEC materials on SRH issues, awareness about the location and services available at the UC by the health facility staff). Addressing these infrastructural barriers may help the adolescents in accessing the SRH services at UCs.

Further, our study flagged that in most of the MC visits, the clients reported a lack of privacy, as several of them reported being easily seen, heard, or distracted by other people in the facility; someone being present throughout the session. Evidence suggests that a lack of privacy and confidentiality experienced by adolescents leads to further distrust for the healthcare providers. Trust is identified as an essential factor impacting the access to- and acceptance for SRH services in successful provider-patient relationships and is one of the most important elements influencing adolescents’ willingness to seek care from the healthcare services [12].

The findings also provide an evidence on the facility-related barriers reported by the counsellors. These include long distance between their home and UCs, limited availability of the transportation facilities and several other infrastructural limitations. One of the challenges faced by counsellors, especially the female counsellor was their perceived inability to manage work and family life due to lack of childcare facilities near workplace. Improving the salaries of the counsellors and providing child-care facilities in the workplace have found to be enabling factors for the women in the workforce to manage both- their work and family life [25].

Our findings also highlighted the need to conduct gender sensitization trainings for counsellors on addressing how to convey the information on sensitive SRH issues to opposite-sex adolescents; and be sensitive on issues such as pre-marital sex, inter-caste and inter-class marriages while providing counselling to the clients. There is a need to provide regular trainings to counsellors to increase their knowledge around SRH matters; and especially around the legalities around some of the health issues. For example, the counsellors demonstrated limited knowledge on abortion laws such as the Medical Termination of Pregnancy Act, complications due to abortion and safe abortion practices. Available literature suggests that lack of knowledge displayed by the health care providers can be a barrier in increasing awareness and utilization of AFHCs [26]. Studies have highlighted that limited knowledge of a health care provider may also result in providing incorrect and incomplete information resulting in poor health outcomes [27]. A qualitative study conducted in Mexico substantiated that failure to provide complete and accurate information about contraceptives to the client led to undermining participants confidence to accurately use contraceptives and further reduced the likelihood of adolescents returning to the facilities and a sense of mistrust towards the health facility and the provider [27].

### Limitations and strengths

The study findings should be read by keeping in mind a few limitations. First, the sample size and the data collected from two districts in Rajasthan may not be representative of the adolescents’ perspective and counsellor’s perspective in the region. Moreover, our findings are based on the interviews of the UC counsellors, however, they are not informed from the perspectives of other types of healthcare providers such as ASHAs, AWWs or medical officers.

Second, due to the limitations associated with the mystery clients’ methodology not undergoing any medical examination or procedures; we are able to explore only a limited number of services provided at the UCs. Additionally, MC scripts were focused only on the topics pertaining to SRH of adolescents which limits our ability to understand if counsellors were able to provide information, services and/or referral for other topics, such as, nutrition. However, we were still able to draw valuable information from the MC’s experiences regarding facility performance and provider’s behaviour. As adolescents were not interviewed, we are unable to confirm whether the services provided by the UC had any impact in terms of increased awareness and improved use of services among adolescents. Third, although there is some chance of the recall bias, to mitigate its effect, the researchers conducting the debriefing of the clients immediately after their visit. Another effort to address this barrier was to send the MCs in pairs to aid their recall about their observations and experiences. To ensure reliability of information shared, researchers conducted the debriefing interviews with the MC and observer separately. Any differences in the observations reported by the pair were discussed and immediately addressed after reaching a consensus from the MC and observer. Fourth, as the debriefing interviews were conducted in the local language (Hindi) and translated in English for analysis, there were risks of not capturing the intended meaning of the information fully. To address this, the interviews were transcribed and translated by professionals with prior experience and fluency in both Hindi and English. Fifth, the WHO framework did not inform the study design. Furthermore, the methodological limitations limited our ability to explore the quality of healthcare services provided at UCs using the complete list of input, process and the output criteria mentioned in the WHO global standards of quality healthcare services for adolescents.

While this resulted in exploring the quality of healthcare using selected criteria from five out of the eight standards, our study also highlights the need of further data and contextual insights to holistically understand how each UC fares against the global standard.

Finally, we would like to highlight some of the strengths of the study. It is important to note that the study contributes to the evidence and research on the quality of SRH services provided to adolescents from both the clients and providers perspective. Second, two key advantages of the MC methodology include its potential for quick feedback, and its association with reduced incidence of bias, such as observer bias and bias from Hawthorne’s effect [11]. Further, the MC methodology is as good a method for monitoring and evaluation because it identifies both strengths and weaknesses of health service delivery and provides the unique perspective of the MCs, the people for whom the service is designed. Lastly, the WHO standards are intended to act as benchmarks against which quality of health care provided to adolescents could be compared, and the criteria developed could help to make the comparisons. It is intended that health service providers use the standards as part of their internal quality assurance mechanisms or as part of an external accreditation process. Our study further underscores the need of examining each of the mentioned criteria within the sociocultural context. For instance, a standard 1 criterion examines whether the ‘counsellors provided age and developmentally appropriate health education and counselling to adolescent clients and inform them about the availability of health, social services and other services. Our study brought forth the need of having specific guidelines that define the age-appropriate information for sensitive SRH topics.

Therefore, this study with its limitations, presents important insights that showcase the existing gaps and possible ways to improve the quality of healthcare services provided at adolescent-friendly health clinics in rural areas in India. This study also presents various insights into the scope and challenges while using the WHO global standard of adolescent healthcare framework in the Indian context.

## Conclusion

The RKSK though aims to increase the awareness about and the service uptake for SRH issues among adolescents, the challenges faced by both the service providers and the adolescents need urgent attention. Some of these challenges are rooted in the sociocultural norms regarding SRH and gender bias, thus, reducing the effectiveness of the adolescent friendly health clinics. Such challenges need to be addressed through comprehensive and inclusive efforts to include community members across different socioeconomic backgrounds, age groups and gender for creating an enabling environment for the adolescents as well as the service providers. Additionally, strengthening the capabilities of the counsellors by training them about the ways in which privacy and confidentiality of the adolescents could be maintained; providing infrastructural and logistical support to them; increasing the visibility of UCs and striving for a dialogue among community members for the need and the use of UCs could mitigate some challenges. Our attempt of using the WHO framework to analyse the data to assess the quality of services at UCs from both the clients and providers perspective, thus, offered an opportunity to not only provide inputs for improving the quality of services provided at AFHCs but could also be helpful in increasing the comparability and the generalizability of our findings with other states and LMICs facing similar challenges.

## Supporting information

S1 Appendix Mystery client scripted scenarios and debrief checklist. (DOCX)

S2 Appendix Performance of Ujala clinics in two districts of Rajasthan state India: Application of the selected standards from ‘WHO Global standard for quality health-care services for adolescents’. (DOCX)

S3 Appendix Debriefing guide for the mystery clients. (DOCX)

S4 Appendix In-depth interview guide for counsellors. (DOCX)
