# Supplementary material for: Assessment of the quality of sexual and reproductive health services delivered to adolescents at Ujala clinics: A qualitative study in Rajasthan, India
Source: PLoS One. 2022 Jan 10;17(1):e0261757. doi: 10.1371/journal.pone.0261757 (PMC8746710; doi:10.1371/journal.pone.0261757)
Supplement: S1 Appendix — (DOCX) [file pone.0261757.s001.docx]

S1 Appendix: Mystery Client Scripted Scenarios and Debrief Checklist

| **Scenarios for Mystery Clients** | **Checklist for Mystery Client Debrief** |
| --- | --- |
| **Script 1: An unmarried girl experiencing menstrual problem:**  A 17-year-old unmarried girl (SUMAN) comes with her younger sister (LALITA, unmarried) to the facility. Suman is feeling shy and nervous while talking about her problem with the counsellor. She has been complaining of severe abdominal pain (during her periods) for the past few months. She thinks that bad blood (from menses) has stuck in her abdomen which might have caused this problem. She is not able to sleep properly and hence, she was referred to this clinic by ASHA. Sometimes, she also gets thick clots in her bleeding due to a bad curse, as suggested by her grandmother. | 1. Did any of the providers whom you met at the clinic tell why you may be experiencing this problem? If yes, who and what did they tell you? 2. Did any of the providers whom you met at the clinic tell you anything about periods in general? If yes, who and what did they tell you? 3. Did any of the providers whom you met at the clinic talk about menstrual hygiene? If yes, who and what did they tell you? 4. When you mentioned about what your grandmother told you, how did they respond? Did any of the providers whom you met at the clinic try to clear your misconception? If yes, who and what did they tell you? 5. Did any of the providers whom you met at the clinic prescribe you any treatment for your problem? If yes, who? 6. If yes, what did they prescribe you? Did they tell you how you should take the prescription? |
| **Script 2: An unmarried boy enquiring about masturbation and nocturnal emission**:  A 16-year-old unmarried boy wants to discuss some issues related to his sexual health with the Ujala counsellor. He has been masturbating for past one year. He got anxious when he recently saw his pants wet (nocturnal emission). He has watched porn a few times. He is very ashamed of this and thinks he has some problem. He has not even discussed this with his friends and family He wants to know what he should do? | 1. Did any of the providers whom you met at the clinic tell you about what is masturbation and nocturnal emission? If yes, who and what did they tell you? 2. When you told them that you are worried that you are having some disease, how did they respond? Did any of them try to clear your misconceptions? If yes, who and what did they tell you? |
| **Script 3: An unmarried female pressurised by boyfriend to have an intercourse:**  A 17-year-old unmarried girl (LAALI) comes with her friend (SUMAN) to the facility. She appears very shy and nervous and says she feels weak and is worried. After which, she sits quietly. After repeated questions by the counsellor, she says that she is in a relationship with a boy in her community since last six months. Last time when he kissed her, she really liked it, and both decided to move further in their relationship. The boy has asked her to have a sexual intercourse a few times after that, but she is worried about getting pregnant before marriage if she goes ahead and has sex with him. She loves and trusts him. She wants to know what she should do. | 1. When you told the provider that your boyfriend is pressurizing you to engage in sex and you don’t know how to handle, how did they respond? Did they advise you about how to handle the situation? If yes, who and what did they tell you? 2. Did any of them talk to you about how you may avoid getting pregnant? If yes, who and what did they say? 3. Did any of them talk to you about your right to say no? If yes, who and what did they say? |
| **Script 4: An unmarried girl seeking information on an unwanted pregnancy:**  An 18-year-old unmarried female (POOJA) wants to get information on ways to avoid unwanted pregnancy. She is accompanied by her friend (MEENA) who begins the conversation with the counsellor as Pooja is feeling scared, shocked and hesitant to talk. Her friend tells the counsellor that Pooja is feeling tired and restless for the past few days. Pooja then talks about her relationship with this boy in her community and that she has been meeting him alone often in the past few months. She is very worried that she has missed her periods and had her last menses 2 months back (the act should make the counsellor ask about her being pregnant) and is worried that she might be pregnant. POOJA hesitates for quite some time to tell the counsellor about why she came to the clinic and kept repeating that the counsellor shouldn’t tell this to anyone | 1. When you narrated your problem of missed period and weakness, how did they respond? What questions did they ask you and who was this person? 2. Did any of them tell you about pregnancy test kit? If yes, who and what did they tell you? 3. Did any of them tell you about abortion? If yes, who and what did they tell you? 4. Did any of them tell you about contraception? If yes, who and what did they tell you? Where to get? How to use? 5. Did they ask about your sexual experiences? If yes, who and what did they ask? |
| **Script 5: An unmarried boy seeking information regarding STIs:**  An 18-year-old unmarried male (RAGHUVIR) wants to access information on Sexually Transmitted Infections (STIs). He is experiencing severe pain and burning sensation while passing urine. His friends are regular with having sexual relations with Female Sex Workers (FSW) and have often made fun of him of not being sexually active till now. He felt pressurized and went to a FSW with his friend. He says that he had unprotected sex a couple of times. He had last sexual intercourse a month back and has been experiencing pain and burning sensation for the past 10 days. He is very worried and had discussed the problem with his friend, who told him that he should visit a doctor. Raghuvir looks confident while talking to the counsellor. He wants to know- what are the reasons for these infections? How can STI be detected/symptoms for STI? Whom should he approach for the treatment? How to cure them? | 1. Did any of the providers take your sexual history? If yes, who? 2. Did any of them tell you about contraceptives? If yes, who and what did they tell you? What you should use, how you should use, where you can get? 3. Did any of the provider tell you about going for any test? If yes, who? What tests? Where? 4. Did any of the provider tell you about partner getting tested? If yes, who, what tests? Where? 5. Did any of them to talk to you about STIs? If yes, who? What did they tell you? 6. Did any of them talk to you about HIV/AIDS? If yes, who and what did they tell you? |
| **Script 6: An unmarried boy requesting condom:**  A 17-year-old unmarried male (MAHESH) wants to get condoms. He is in a relationship with a girl for past six months. He is sexually active and has heard about condoms but had never used them before. He really likes the girl and wants to marry her, although they belong to different caste. He left his studies after 9th grade and now, he often works as construction labourer. He has come to the clinic to find more about condoms. |  |
| **Common Questions asked to all the participants:** | 1. Time taken to reach the Ujala clinic? 2. Directional signs to the UC? 3. Registered required and process? 4. Behavior of the hospital staff? 5. Description of the waiting room outside UC? 6. Description of the UC? 7. IEC material displayed inside and outside the UC? 8. Behaviour of the counsellor? 9. Quality of the health facility? |
