# Supplementary material for: Assessment of the quality of sexual and reproductive health services delivered to adolescents at Ujala clinics: A qualitative study in Rajasthan, India
Source: PLoS One. 2022 Jan 10;17(1):e0261757. doi: 10.1371/journal.pone.0261757 (PMC8746710; doi:10.1371/journal.pone.0261757)
Supplement: S2 Appendix — (DOCX) [file pone.0261757.s002.docx]

**S2 Appendix:**

**Performance of Ujala Clinics in two districts of Rajasthan state India: Application of the selected standards from ‘WHO Global standard for quality health-care services for adolescents’**

**Standard 1- Adolescent health literacy**

Global Standard 1 for quality health-care services for adolescents calls for health facilities to put in systems to ensure that adolescents are knowledgeable about their own health, and they know where and when to obtain health services (WHO, 2015). We probed the counsellors and the MCs on the following mentioned criteria for standard 1.

|  | **Intervention** | | **Control** | |
| --- | --- | --- | --- | --- |
| **STANDARD 1 CRITERIA** | **UC I** | **UC II** | **UC I** | **UC II** |
| **INPUT** |  | | | |
| **The health facility has a signboard that mentions operating hours** | Directional signboards both inside and outside the facility. Name, operating hours and contact of the counsellor mentioned. | Directional signboards only inside the facility. Name, operating hours and contact of the counsellor mentioned. | No directional signboards present, however, operating hours mentioned on the board. | Directional signboards only inside the facility. No operating hours mentioned. |
| **The health facility has in the waiting area up-to-date information, education and communication materials specifically developed for adolescents.** | No specific waiting area for UC. Benches and chairs kept in the corridor of the clinic. IEC material on the following topics was displayed on the walls either inside or outside the UC. [explored only for the topics pertaining to sexual and reproductive health] | No waiting area outside the UC. IEC material on the following topics was displayed on the walls either inside or outside the UC. [explored only for the topics pertaining to sexual and reproductive health] | No waiting area outside the UC. IEC material on the following topics was displayed on the walls either inside or outside the UC. [explored only for the topics pertaining to sexual and reproductive health] | No waiting area outside the UC. IEC material on the following topics was displayed on the walls either inside or outside the UC. [explored only for the topics pertaining to sexual and reproductive health] |
| *Menstrual Hygiene* | ✓ | ✓ | ✓ | ✓ |
| *Contraceptives and family planning* | ✓ | ✓ | ✓ | ✓ |
| *Sex education* | X | X | X | ✓ |
| *Child marriage and early pregnancy* | X | ✓ | X | X |
| *Domestic and sexual violence* | X | X | ✓ | ✓ |
| *Substance abuse* | X | ✓ | ✓ | ✓ |
| *Nutrition and healthy diet* | X | ✓ | X | ✓ |
| *HIV/AIDS* | X | X | ✓ | X |
| *Information on RKSK & toll-free helpline number (104/108)* | ✓ | ✓ | ✓ | ✓ |
| **Health-care providers have competencies to provide health education to adolescents and to communicate about health and available services (health, social and other services).** | The provider had technical competencies to provide health information, counselling; however, input regarding busting the myths on menstruation and nocturnal emission seemed inadequate | The provider had technical competencies to provide health information, counselling; however, it seemed to lack the competency regarding busting myths on menstruation and nocturnal emission | Yes, the provider had technical competencies to provide health information, counselling and in busting myths on menstruation and nocturnal emission | Yes, the provider had technical competencies to provide health information, counselling and in busting myths on menstruation and nocturnal emission |
| **The health facility has outreach workers that are trained to conduct health education for adolescents in the community.** | - The outreach activities are conducted by the counsellors themselves, who are often accompanied by the ANM and a lab technician. - UC had outreach workers specifically trained to conduct health education for adolescents in the community. | | | |
| **The health facility has a plan for outreach activities and/or involvement of outreach workers in activities to promote health and increase adolescents’ use of services** | - Every UC had a plan to conduct the outreach activity [6 outreach programs per month] in the schools and Anganwadi centres in nearby villages. - UC had a plan for outreach workers to be involved in activities to promote health and increase adolescents’ use of services | | | |
| **PROCESS** |  | | | |
| **Health-care providers provide age and developmentally appropriate health education and counselling to adolescent clients and inform them about the availability of health, social services, and other services** | When presented with different case scenarios, the counsellors shared the advice that they would provide to adolescents. However, none of the counsellors specifically mentioned how their messaged, and advice would differ for adolescents with different age and developmental milestones. | | | |
| **Outreach activities to promote health and increase adolescents’ use of services are carried out according to the health facility’s plan.** | - Every UC conducted the outreach activity [6 outreach programs per month] in the schools and Anganwadi centres in nearby villages. - UC involved outreach workers in activities to promote health and to increase adolescents’ use of services | | | |
| **OUTPUT** |  | | | |
| Adolescents are knowledgeable about health | As we use the trained mystery clients’ visits as the methodology, we are unable to assess these output criteria. | | | |
| Adolescents are aware of what health services are being provided, where and when they are provided and how to obtain them |  |  |  |  |

**Standard 3- Appropriate package of services**

Global standard 3 for quality health-care services for adolescents expects the health facilities to provide a package of information, counselling, diagnostic, treatment, and care services that fulfils the needs of all adolescents in the clinic and through referral linkages and outreach (WHO, 2015). We present our findings on the selected criteria, as shown below, based on our findings from counsellors’ IDIs and MC debriefing interviews.

|  | **Intervention** | | **Control** | |
| --- | --- | --- | --- | --- |
| **STANDARD 3 CRITERIA** | **UC I** | **UC II** | **UC I** | **UC II** |
| **INPUT** |  | | | |
| Policies and procedures are in place that describe the referral system to services within and outside the health sector, including provisions for transition care for adolescents with chronic conditions | Yes | Yes | Yes | Yes |
| **PROCESS** |  | | | |
| Service providers refer adolescents to the appropriate service and level of care according to local policies and procedures and follow the policies for transition care. | Yes. Counsellor mentioned that they refer adolescents to doctor for any treatment of a disease.  Data from one MC debriefing session indicated that the counsellor referred the MC to the doctor for the symptoms of STIs. | Yes. Counsellor mentioned that they refer adolescents to a doctor for mostly nocturnal emission related issue.  Data from two MC debriefing session indicated that the counsellor referred the MC to the doctor for advice regarding masturbation and  nocturnal emission; and STI | Yes. Counsellor mentioned that they have referred adolescent to a doctor/urologist for mostly nocturnal emission related issue.  Data from one MC debriefing session indicated that the counsellor advised the MC to refer to a doctor or a psychologist regarding masturbation and nocturnal emission | Yes. Counsellor mentioned referring an adolescent to a doctor for a for a sexual health related issues like experiencing burning sensation while urinating |
| **OUTPUT** |  | | | |
| The health facility provides a package of health services that fulfils the needs of all adolescents, in the facility and/ or through referral linkages and outreach | Yes | Yes | Yes | Yes |

**Standard 4- Providers’ competencies**

Global standard 4 for quality adolescent care expects the health-care provider to demonstrate the technical competence required to provide effective health services to adolescents, provide privacy and confidentiality of information and maintain a non-judgemental attitude and respect towards the clients. (WHO, 2015). Below findings based on counsellor IDIs and MC interviews present the nuances of the providers’ competencies.

|  | **Intervention** | | **Control** | |
| --- | --- | --- | --- | --- |
| **STANDARD 4 CRITERIA** | **UC I** | **UC II** | **UC I** | **UC II** |
| **INPUT** |  | | | |
| **Health-care providers and support staff of the required profile are in place*** | No, only 1 male counsellor available [No female counsellor] | No, only 1 female counsellor available [no male counsellor] | No, only 1 male counsellor available [no female counsellor] | No, only 1 male counsellor available [no female counsellor] |
| **Health-care providers have the technical competencies necessary to provide the required package of services** | The provider had technical competencies to provide health information, counselling; however, input regarding busting the myths on various topics of SRH seemed inadequate.  The provider also had the competency in supplying and demonstrating how to use the required health commodities [iron tablets, sanitary napkins, condoms, pregnancy kit and pain relief tablets]. | The provider had technical competencies to provide health information, counselling; however, it seemed to lack the competency regarding busting myths on various topics of SRH.  The provider also had the competency in supplying and demonstrating how to use the required health commodities [iron tablets, sanitary napkins, condoms, pregnancy kit and pain relief tablets]. | Yes, the provider had technical competencies to provide health information, counselling and in busting myths on topics related to SRH.  The provider also had the competency in supplying and demonstrating how to use the required health commodities [iron tablets, sanitary napkins, condoms, pregnancy kit and pain relief tablets]. | Yes, the provider had technical competencies to provide health information, counselling and in busting myths on topics related to SRH.  The provider also had the competency in supplying and demonstrating how to use the required health commodities [iron tablets, sanitary napkins, condoms, pregnancy kit and pain relief tablets]. |
| **Health-care providers have been trained/sensitized on the importance of respecting the rights of adolescents to information, privacy, confidentiality, and of the health care that is provided in a respectful, non-judgemental, and non-discriminatory manner** | All counsellors mentioned receiving six days training programme for adolescent health counsellors on RKSK program by the department of health and family welfare and by an external organization on strengthening counselling skills of the counsellors | | | |
| **Providers’ obligations and adolescents’ rights are clearly displayed in the health facility** | When asked about the materials and messages displayed in UCs, none of the MCs reported any display of providers’ obligations and adolescents’ rights in any UC. | | | |
| **Up-to-date decision support tools (guidelines, protocols, algorithms) that cover topics of clinical care in line with the package of services are in place** | All the counsellors mentioned being given the support materials to offer the package of services and counselling to adolescents. | | | |
| **A system of supportive supervision is in place to improve health-care providers’ performance** | Yes | Not asked | Yes | Yes |
| **PROCESS** |  | | | |
| **Health-care providers follow evidence-based guidelines and protocols in delivering care to adolescents** | The narratives of some of the counsellors indicated that they were provided the support materials during their training which would help them in offering the package of services and counselling to adolescents. However, our debriefing sessions with MCs indicated that the counsellors did not physically refer to any support materials while providing the counselling services. | | | |
| **Health-care providers and support staff relate to adolescents in a friendly manner, and respect their rights to information, privacy, confidentiality, non-discrimination, non-judgemental attitude, and respectful care** | Although all the four counsellors were reported to be friendly with the MCs, at several instances their awkwardness in discussing the sensitive topics; their potential bias for caste, and marital status was evident. | | | |
| **OUTPUT** |  | | | |
| **Adolescents receive effective health services and Adolescents receive accurate, age- appropriate and clear information to facilitate informed choice** | As narrated by the MCs, although health information on specific health topics was discussed, it was evident that dietary advice was often coupled with a generalized life advice. For example, advice of ‘*reading religious, historical or spiritual books before going to bed*’, as reflected in the quote below.  “*…He advised me to get up in the morning, jog, and drink lukewarm water before going to the washroom. Take hot milk and eat more green vegetables, avoid junk food and instead eat home-cooked food only, read some religious, historical or spiritual books before going to sleep, can attempt rope skipping, it will be good for my health and spend more time with friends”.*  MC, male, [Script on masturbation and nocturnal emission] | As narrated by the MCs, health information was provided but it seemed to reflect awkwardness of the counsellor in discussing a few topics. For instance, one MC reported that upon asking for more details of the condom, the female counsellor asked him to refer to the box of condoms and asked the MC whether he was literate. He also reported that the counsellor left the clinic while the conversation on condoms was ongoing.  MC, female, [Script on masturbation and nocturnal emission] | Although the counsellor seemed to provide health information on some topics, the counsellor seemed misinformed about masturbation. The MC with a script on masturbation was provided with some information about what masturbation means but was also given a misleading referral to a psychologist and was asked to “*plant a tree”.*  MC, male, [script on masturbation and nocturnal emission] | Based on the experiences shared by the MCs, basic health information was provided by the counsellor. One male MC who narrated a script on nocturnal emissions and asked if masturbation is a disease was advised,  *“You can keep your phones away. This age is such that we have a desire to watch a specific type of videos that come on our phones, like blue films. He asked if I watched them, and I did not reply at first. He told me that it is wrong to watch such videos and we should keep our phones away as much as possible as it has a negative influence.”*  MC, male, [script on masturbation and nocturnal emission] |
| **Adolescents receive services in a friendly, supportive, respectful, non-discriminatory, and non-judgemental manner, and know their rights in health care.** | - Warm, friendly, and sensitive - Non-judgemental - Explained everything in detail and answered all the questions - Respectful towards others - Gave his contact number and asked to call if needed | - Friendly and polite - Busy on the phone and disinterested - Was in a hurry and rushed the conversation - Displayed lack of knowledge and shared generic information - Did not give opportunities to ask any questions - Showed judgemental attitude towards inter-caste marriage | - Knowledgeable, explained everything in detail - Warm and friendly as per one MC - Gave adequate time - Felt intimidated, as described by one MC (Script 4) - Used abusive words (Script 5) - Showed judgemental and biased attitude towards pre-marital sex (Script 3, 4 & 6) | - Polite and friendly - Understanding, patient and explained everything in detail - Respectful and non-judgemental - Did not give a lot of information, was hesitant in talking and seemed shy |

**Standard 5-Facility characteristics**

Global standard 5 for quality adolescent care calls for the health facility to provide convenient operating hours, a welcoming and clean environment and maintain privacy and confidentiality; provides for medicines, supplies and technology needed to ensure effective service provision to adolescents (WHO, 2015. We analysed the data from MC debriefing sessions and from the IDIs with the counsellors to assess the selected criteria for this standard.

|  | **Intervention** | | **Control** | |
| --- | --- | --- | --- | --- |
| **STANDARD 5 CRITERIA** | **UC I** | **UC II** | **UC I** | **UC II** |
| **INPUT** |  | | | |
| **The facility has basic amenities** | Most of the MCs and observers reported overall cleanliness from inside and outside.    The experience of using toilets was mixed as most of the MCs who used the toilet reported it to be unclean without water.  All the MCs reported an availability of drinking water at the facility. | The reports regarding the overall cleanliness of the facility from inside and outside were mixed, and maximum reported it to be unclean.  Reports of half of the MCs who visited the toilet in the facility mentioned it to be non-functional.  Only one MC mentioned the availability of drinking water. Rest MCs reported it to be unavailable in the facility. | Only one MC mentioned the overall cleanliness of the facility to be poor. Rest MCs reported it to be clean.  Only half of the MCs visited the toilet in this facility, out of which most mentioned it to be non-functional  All the MCs reported an availability of drinking water at the facility. | Most of the MCs who reported about the overall cleanliness of the clinic mentioned it to be clean.  Except for one, all the MCs visited the toilet in the facility and reports were mixed.  Half of the MCs when visited the clinic, found the drinking water to be available and the remaining half found it to be unavailable. |
| **Policies and procedures to protect the privacy and confidentiality of adolescents are in place. Both health-care providers and support staff know them as well as their own roles and responsibilities.** | Yes | Yes | Yes | Yes |
| **PROCESS** |  |  |  |  |
| **Health-care providers offer consultations during hours that are convenient to adolescents in local communities, with or without an appointment** | Yes, all MC visits were made without an appointment | | | |
| **Health-care providers and support staff follow policies and procedures to protect the privacy and confidentiality of adolescents** | All counsellors were aware of the importance of maintaining privacy and confidentiality while providing SRH services to adolescents. However, the debriefing interviews with most of the MCs reported a breach in their privacy and confidentiality due to someone else being present in the session and/or frequent visits by strangers | | | |
| **OUTPUT** |  | | | |
| **The health facility has convenient operating hours, appointment procedures and waiting times kept to a minimum.** | Counsellor mentioned Monday to Saturday 9:00 am-4:00 pm as operating hours. Days when outreach activities are conducting, clinic timings are from 9:00 to 12:00 pm  No procedure for taking an appointment was required. | Counsellors mentioned 9:00am-4:00 pm as the operating hours (counsellor reached by 10 am due to lack of transportation facilities from home to the UC).  No procedure for taking an appointment was required. | Counsellors mentioned 9:00am-4:00 pm as the operating hours (counsellors reached an hour early to minimize waiting time).  No procedure for taking an appointment was required. | Counsellors mentioned 9:00am-4:00 pm as the operating hours.  No procedure for taking an appointment was required. |
| **Adolescents always receive private and confidential health care during the consultation process.** | Our IDIs with counsellors indicated that all the four counsellors were aware of the importance of maintaining privacy and confidentiality while providing SRH services and of building a rapport to make the adolescents comfortable for discussing their health issues.  However, in many visits made by the MCs, the clients reported a breach in their privacy and confidentiality as MCs reported disturbance from the staff and other patients visiting the clinic during the counselling sessions. | | | |
| **The facility has the equipment, medicines*, supplies* and technology needed to ensure effective service provision to adolescents**  **asked counsellors and MCs if medicines and supplies were provided to adolescents* | Yes | Yes | Yes | Yes |

**Standard 6- Equity and non-discrimination**

Global standard 6 for equity and non-discrimination expects the health care provider to provide quality services to all adolescents irrespective of their ability to pay, age, sex, marital status, education level, ethnic origin, sexual orientation, or other characteristics. (WHO 2015). We analysed the narratives of the mystery client and in-depth interviews of the counsellors to assess the behaviour of the counsellors, in terms of demonstrating friendly, non-judgemental, and respectful attitude to all adolescents while providing counselling services.

|  | **Intervention** | | **Control** | |
| --- | --- | --- | --- | --- |
| **STANDARD 6 CRITERIA**** | **UC I** | **UC II** | **UC I** | **UC II** |
| **PROCESS** |  | | | |
| **Health-care providers and support staff demonstrate the same friendly, non-judgemental, and respectful attitude to all adolescents, regardless of age, sex, marital status, sexual orientation, cultural background, ethnic origin, disability, or any other reason.** | Debriefing with the MCs indicated that healthcare provider was friendly, non-judgemental, respectful to all MCs regardless of age, sex, marital status. | Debriefing with the MCs indicated that healthcare provider was friendly, respectful to all MCs regardless of age, sex, marital status.  However, it was found that the counsellor displayed caste-related bias towards male MCs. | Debriefing with the MCs indicated that healthcare provider was friendly to al MCs regardless of age, marital status. However, demonstrated a biased attitude towards female MCs with regards to Script 3, 4 and 6. | Debriefing with the MCs indicated that healthcare provider was friendly, non-judgemental, respectful to all MCs regardless of age, sex, marital status. |
