# Supplementary material for: Assessment of the quality of sexual and reproductive health services delivered to adolescents at Ujala clinics: A qualitative study in Rajasthan, India
Source: PLoS One. 2022 Jan 10;17(1):e0261757. doi: 10.1371/journal.pone.0261757 (PMC8746710; doi:10.1371/journal.pone.0261757)
Supplement: S3 Appendix — (DOCX) [file pone.0261757.s003.docx]

S3 Appendix: Debriefing guide for the mystery clients

Debriefing guide for the mystery clients

रहस्य ग्राहकों के लिए डीब्रीफिंग गाइड

Identification

| **ftyk dksM@**  DISTRICT CODE |  |
| --- | --- |
| **[k.M dksM@**  BLOCK CODE |  |
| **lqfo/kk dsUnz dksM@**  FACILITY CODE |  |
| **iznkrk /**Provider No. | **mtkyk Dyhfud** /Ujala Clinic……….1  **vksihMh** /OPD………………………..2 |
| **feLVªh DykbaV dksM@**Mystery Client Code/ प्रेक्षक **dksM** Observer Code | **feLVªh DykbaV** /Mystery Client ……..1  प्रेक्षक /Observer………..…………….2 |
| **feLVªh DykbaV dh mez@** Age of the mystery client/ प्रेक्षक **dh mez** /Age of observer |  |
| **feLVªh DykbaV dk fyax@**Sex of the mystery client | **iq:”k@**Male ……………………..1  **efgyk@**Female………………….2 |
| **fuHkk;k x;k ifjn`’;@**  Scenario enacted | **ekgokjh leL;kvksa dk vuqHko djus okyh ,d vfookfgr efgyk@**  An unmarried female experiencing menstrual problems …………….…..….1  **gLreSFkqu vkSj LoIunks”k ds ckjs esa iwNrkN djus okyk ,d vfookfgr yMdk@**  An unmarried boy enquiring about masturbation and nocturnal emission ….2  **lkFkh }kjk ;kSu laca/k cukus ds ncko dk lkeuk dj jgh ,d vfookfgr efgyk@**  An unmarried female pressured by partner to have intercourse ……………..3  **vupkgh xHkkZoLFkk ij tkudkjh [kkstus okyh ,d vfookfgr efgyk@**  An unmarried female seeking information on unwanted pregnancy………….4  **;kSu lapkfjr laØe.k ds laca/k esa tkudkjh [kkstus okyk ,d vfookfgr iq:”k@**  An unmarried male seeking information regarding STIs…………………..…5  **daMkse ekaxus okyk ,d vfookfgr iq:”k@**  An unmarried male requesting condom…………………..…………………..6 |
| **lk{kkRdkj dh rkjh[k@**  DATE OF INTERVIEW | _____________________ |

**feLVªh DykbaV fMczhQ ds fy, lk{kkRdkj funsZf’kdk@**

**Interview guide for Mystery Client Debrief**

**ueLdkj] eSa ----------------- gwa vkSj eSa vkils vkidh feLVªh DykbaV foftV ds ckjs esa ckr djuk pkgwaxkA eSa vkidks Hkjkslk fnykuk pkgwaxk fd lHkh tokcksa dks xksiuh; j[kk tk;sxk vkSj lk{kkRdkj esa yxHkx ,d ?kaVk yxsxkA vkids }kjk iznku dh tkus okyh dksbZ egRoiw.kZ tkudkjh NwV uk tk;sa blls cpko ds fy, eSa gekjh ckrphr dks fjdkMZ djuk pkgwaxkA vkils fuosnu gS fd d`i;k mtkyk Dyhfud ij viuh foftV ds voyksdu vkSj vuqHko ls lacaf/kr izR;sd fooj.k dks crk;saA**

Namaskar, I am ________ and I would like to talk to you about your mystery client’s visit. I would like to assure you that all responses will be kept confidential and the interview should take about an hour. I would like to record our conversation to avoid missing out on any important information that you may provide. You are requested to please share every detail related to your observation and experience from your visit to the Ujala clinic.

gekjs ‘kq: djus ls igys D;k vkids dksbZ loky gSa\@

Do you have any questions for us before we begin?

**ge ,sls ?kVukØeksa ds lkFk viuh ckrphr ‘kq: djuk pkgsaxs tks vkids vLirky igqapus tgka mtkyk fDyhfud fLFkr gS vkSj tc vki vLirky ls okil vk;s rd ds chp ds le; esa tks dqN Hkh gqvk gSA@**We would like to start our discussion with a timeline of events that happened between the time you reached the hospital where UJALA clinic is located and the time you left the hospital

**vki vLirky fdl le; ij igqaps\@**What time did you reach the hospital?

**vLirky igqapus ds ckn] vkius lcls igys D;k fd;k\ ogka vki fdlls feys\ vkius blds fy, fdruk le; fcrk;k\@**After reaching the hospital, what did you do first? Whom did you meet there? How much time did you spend for this?

**mlds ckn vkius D;k fd;k\ vki blds fy, fdl ls feys\ vkius blds fy, fdruk le; fcrk;k\@**What did you do afterwards? Who did you meet for this? How much time did you spend for this?

**¼d`i;k DykbaV }kjk vLirky esa fcrk;s x;s dqy le; ds ckjs esa lkjh tkudkjh ysa] t:jh ugha fd ;g mtkyk Dyhfud ij gh gks] lHkh ?kVuk;sa tks bl vof/k esa gqbZ vkSj lHkh yksx ftuls og bl vof/k ds nkSjku feyk½@**

(PLEASE ACCOUNT FOR THE TOTAL TIME THAT THE CLIENT SPENT AT THE HOSPITAL, NOT NECESSARILY AT THE UJALA CLINIC, ALL THE EVENTS THAT HAPPENED DURING THIS PERIOD AND ALL THE PEOPLE WHOM THE CLIENT MET DURING THIS PERIOD)

| **Q No.** | **Question and Filters** | **Code/Response Categories** | **Skip To** |
| --- | --- | --- | --- |
| **1** | **D;k vkidks mtkyk Dyhfud ¼v’kZ Dyhfud½ vklkuh ls fey x;k\**  Did you find the Ujala Clinic (ARSH Clinic) easily? | **gka@**Yes…………………………….1  **ugha@**No…………………………....2 |  |
| **1a** | **mtkyk Dyhfud dks vklkuh ls [kkstus esa fdlus vkidh enn dh\@**  What/who helped you to locate the UJALA clinic easily?  **vkidks ,slk D;ksa yxk fd mtkyk Dyhfud dks [kkstuk eqf’dy Fkk\@**  Why did you feel that it was difficult to locate the UJALA clinic? | ________________________________  ________________________________  ________________________________  ________________________________ |  |
| **2** | **D;k vLirky ds ckgj mtkyk Dyhfud dgka gS ;s crkus ds fy, dksbZ fn’kklwpd ladsr Fks\@**  Were there any directional signs OUTSIDE the hospital building indicating the UJALA clinic’s location? | **gka@**Yes…………………………….1  **ugha@**No…………………………....2 |  |
| **2a** | **;fn gka] rks mu lkbucksMZ ij D;k fy[kk@ fn[kk;k gqvk Fkk\@**  If yes, what was written/shown on the signboard? | ________________________________  ________________________________  ________________________________  ________________________________ |  |
| **3** | **D;k vLirky ds vanj mtkyk Dyhfud dgka gS ;s crkus ds fy, dksbZ fn’kklwpd ladsr Fks\@**  Were there any directional signs INSIDE the hospital building indicating the UJALA clinic’s location? | **gka@**Yes…………………………….1  **ugha@**No…………………………....2 |  |
| **3a** | **;fn gka] rks mu lkbucksMZ ij D;k fy[kk@ fn[kk;k gqvk Fkk\@**  If yes, what was written/shown on the signboard? | ________________________________  ________________________________  ________________________________  ________________________________ |  |
| **4** | **mtkyk Dyhfud ij LokLF; ns[kHkky iznkrk ¼lykgdkj½ ls vkids feyus ls igys D;k dksbZ vkSipkfjdrk,sa Fkh tks vkius iwjh dh Fkh\@**Were there any formalities you had to go through before you see the health care provider (counsellor) at the UJALA clinic? | **gka@**Yes…………………………….1  **ugha@**No…………………………....2 | Q 8 |

| **Q No.** | **Question and Filters** | **Code/Response Categories** | **Skip To** |
| --- | --- | --- | --- |
| **5** | **os vkSipkfjdrk,sa D;k Fkh\@**What were those formalities? | **eq¶r vksihMh iathdj.k@**Free OPD registration………………A  **vksihMh iathdj.k dkmaVj ij ‘kqYd pqdk;k@**Paid fees at OPD registration counter……………………………..B  **vU; vLirky deZpkjh dks vkus ds fy, dkj.k crkuk iMk@**Had to answer to other hospital staff  the reason for coming………..…….C  **vU; ¼crk;sa½@**  Other (specify)_________________X |  |
| **6** | **;fn iathdj.k izfØ;kvksa ls gksdj xqtjuk iMk] rks iathdj.k MsLd ij deZpkjh us vkids lkFk dSlk O;ogkj fd;k\@**If required to go through registration process, how did the staff at the registration desk behave with you?  **;fn mtkuk Dyhfud ij tkus ls igys vU; vLirky deZpkfj;ksa ds lokyksa ds tokc nsus dh t:jr gqbZ] rks deZpkjh us vkids lkFk dSlk O;ogkj fd;k\@**If required to answer the queries of other hospital staff before going to the UJALA clinic, how did the other staff behave with you?  **vfHkoknu] muls ‘kq:vkrh iwNrkN] mudh izfrfØ;k,sa vkfn ds ckjs esa iwNsa@**Probe about greetings, initial enquiries from them, their expressions etc | ________________________________  ________________________________  ________________________________  ________________________________  ________________________________  ________________________________ |  |
| **6a** | **os dkSu lh tkudkfj;ka gSa tks iathdj.k MsLd@ vU; vLirky deZpkfj;ksa us vkils yh\@**What are the details that staff at the registration desk/other hospital staff collected from you? |  |  |
| **7** | **iathdj.k MsLd ij deZpkfj;ksa dk@ vU; vLirky deZpkfj;ksa dk joS;k@O;ogkj dSlk Fkk ftlus vki mtkyk Dyhfud ij tkus ls igys feys\ D;k vki dgsaxs nksLrkuk] vlH;@nksLrkuk ugha ;k cl fu”i{k\@**How was the attitude of the staff at the registration desk/ other hospital staff whom you met before going to the UJALA clinic? Would you say friendly, rude/not friendly or just neutral? | **nksLrkuk@**  Friendly……………………………….1  **vlH;@nksLrkuk ugha@**  Rude/not friendly ..…………………..2  **cl fu”i{k@**  Just neutral…………………………...3 |  |
| **8** | **lykg ysus ds fy, vkidks dgka tkus ds fy, dgk x;k Fkk\ ¼mtkyk Dyhfud dh iqf”V djsa ;fn ogka dksbZ lkbucksMZ Fkk@ ml dejs ds vanj dsoy fd’kksj@fd’kksfj;ka DykbaV~l gh Fks½@**Where were you directed to go for the consultation? (confirm Ujjala clinic only if there was signboard/only adolescent clients inside that room) | **mtkyk Dyhfud@**  Ujala Clinic……………………….1  **lkekU; vksihMh okyk dejk@**  General OPD room…………..….2  **vU; ¼d`i;k crk;sa½@**  Others (please specify)______________ | Q10 |

| **Q No.** | **Question and Filters** | **Code/Response Categories** | **Skip To** |
| --- | --- | --- | --- |
| **9** | **D;k vki ml dejs ds ckjs esa o.kZu dj ldrs gSa tgka vkius ijke’kZ fy;k Fkk\ ¼fLFkfr] vyx txg@dejk] ijke’kZ d{k ds Hkhrj ekgkSy ds fy, iwNsa & ogka dkSu&dkSu Fks\ fdrus yksx\ ogka dkSu&dkSu lh lqfo/kk,sa Fkh\ dksbZ vkbZbZlh lkefxz;ka n’kkZbZ xbZ Fkh\ LoPNrk\ futrk vkfn\½@**Can you describe the room where you had the consultation? (Probe for location, dedicated space/room, ambience inside the consulting room – who all were there? How many people? What facilities were there? Any IEC materials displayed? Cleanliness? Privacy etc)? | ________________________________  ________________________________  ________________________________  ________________________________  ________________________________  ________________________________ |  |
| **9a** | **If coded only 2 in Q8 then go to Q12a, else continue** | |  |
| **10** | **D;k mtkyk Dyhfud ds ckgj dksbZ izrh{kk djus dk ,fj;k Fkk\@**Was there a waiting area outside the Ujala Clinic? | **gka@**Yes…………………………….1  **ugha@**No…………………………....2 | Q11 |
| **10a** | **;fn gka] rks D;k vki izrh{kk ,fj;k ds ckjs esa o.kZu dj ldrs gSa\ ¼izrh{kk ,fj;k dh fLFkfr] ekgkSy ds fy, iwNsa & dsoy fd’kksjksa&fd’kksfj;ksa@lHkh ds fy,\ ogka fdrus yksx Fks\ ogka dkSu lh lqfo/kk,sa Fkh\ dksbZ n’kkZbZ xbZ lkefxz;ka\ LoPNrk\ fd’kksjksa&fd’kksfj;ksa ds fy, futrk vkfn½\@**If yes, can you describe the waiting area? (Probe for location, ambience of the waiting area– for only adolescents/ all? How many people? What facilities were there? Any IEC materials displayed? Cleanliness? Privacy for adolescents etc)? | ________________________________  ________________________________  ________________________________  ________________________________  ________________________________  ________________________________ |  |
| **11** | **;fn izrh{kk ,fj;k ;k lykg d{k ds Hkhrj dksbZ vkbZbZlh lans’k@lkefxz;ka crkbZ xbZ] rks fdl izdkj dh lkefxz;ka n’kkZbZ xbZ Fkh\ mu lkefxz;ksa esa dkSu ls lans’k izlkfjr fd;s x;s Fks\@**If reported IEC messages/ materials in the waiting area or inside the consulting room, what sort of materials were displayed? What were the messages conveyed in those materials? | ________________________________  ________________________________  ________________________________  ________________________________  ________________________________  ________________________________ |  |
| **12** | **D;k vkidks yxrk gS fd’kksj&fd’kksfj;ka mu lkefxz;ka@lans’kksa dks i<+uk ilan djsaxs\ vkidks ,slk D;ksa yxrk gS\ ¼mi;qDr lans’k] bLrseky dh xbZ Hkk”kk] bLrseky dh xbZ rLohjsa] ubZ tkudkjh vkfn ij iwNsa½@**Do you think adolescents would like to read those materials/ messages? What makes you think so? (probe on relevant messaging, language used, images used, new information, etc.) | ________________________________  ________________________________  ________________________________  ________________________________  ________________________________  ________________________________ |  |
| **Q No.** | **Question and Filters** | **Code/Response Categories** | **Skip To** |
| **12a** | **vkius eq>s crk;k fd vki LokLF; dsUnz ij ------------ ls feys( vkb;s ------- ¼igyk O;fDr ftlls DykbaV iathdj.k@’kq:vkrh iwNrkN ds ckn feyk vkSj O;fDr dk inuke fy[ksa ;fn izfrHkkxh dks irk gS vkSj LokLF; dsUnz ij feys lHkh iznkrkvksa ls iz39 rd nksgjk;sa½ ls ‘kq: djrs gSa@**You told me that you met ……..at the facility; let’s start with …. (first person whom client met after registration/the initial enquiry and write the person’s designation if the respondent knows and repeat upto Q39 for all the providers met at the facility | ________________________________  ________________________________  ________________________________  ________________________________  ________________________________  ________________________________ |  |
| **13** | **D;k ;g O;fDr iq:”k Fkk ;k efgyk Fkh\@**Was this person male or female? | **iq:”k@**Male………………………1  **efgyk@**Female……………………2 |  |
| **14** | **bl O;fDr us vkids lkFk dSlk O;ogkj fd;k\@**how did this person behave with you?  **vfHkoknu] ckrphr ‘kq: djus ds fy, muds }kjk iwNs x;s lokyksa] muds Hkkoksa vkfn ds ckjs esa iwNsa@**Probe about greetings, questions they asked to start of the conversation, their expressions etc | ________________________________  ________________________________  ________________________________  ________________________________  ________________________________  ________________________________ |  |
| **15** | **D;k ogka vU; yksx ¼DykbaV~l@vU; iznkrk tks dsl ls lacaf/kr ugha½ ekStwn Fkk tgka vkius bl O;fDr ds lkFk ckrphr dh Fkh\@**Were other people (clients/other providers not related to the case) where you interacted with this person? | **gka@**Yes…………………………….1  **ugha@**No…………………………....2 |  |
| **16** | **D;k bl O;fDr ds lkFk gqbZ vkidh ckrphr dks vU; yksxksa us lquk@ns[kk\@**Could other people have heard/ seen your conversation with this person? | **ns[kk gks ldrk gS@**  Could have seen………………….1  **lquk gks ldrk gS@**  Could have heard………………….2  **lquk vkSj ns[kk gks ldrk gS@**  Could have heard and seen………..3  **dksbZ ugha@**None …………………4 |  |
| **17** | **D;k bl O;fDr ds lkFk ckr djus esa fd’kksj@fd’kksfj;ka lgt eglwl djsaxs\ ,slk D;ksa\@**Would adolescents feel comfortable talking with this person? Why so? | ________________________________  ________________________________  ________________________________  ________________________________  ________________________________  ________________________________ |  |
| **18** | **D;k lykg ds nkSjku bl O;fDr us dksbZ tYnckth fn[kkbZ\@**Did this person rush through the consultation? | **gka@**Yes…………………………….1  **ugha@**No…………………………....2 |  |
| **Q No.** | **Question and Filters** | **Code/Response Categories** | **Skip To** |
| **19** | **D;k vkius mls LokLF; leL;k ds ckjs esa crk;k ftlds fy, vki mtkyk Dyhfud ij bl O;fDr ls feyus x;s Fks\@**Did you share the health concern for which you visited the UJALA clinic with this person? | **gka@**Yes…………………………….1  **ugha@**No…………………………....2 |  |
| **20** | **tc vkius viuh leL;k ds ckjs esa crk;k Rkks lykgnkrk us dSls tokc fn;k\@**How did the counsellor respond when you narrated your problem?  **bl O;fDr }kjk iwNs x;s iz’uksa ds ckjs esa iwNsa( fVIif.k;ka@voyksdu@bl O;fDr }kjk nh xbZ lykg( mlds psgjs ij Hkko½@**Probe about questions this person asked; comments/observations/ advice this person made; his/her facial expressions) | ________________________________  ________________________________  ________________________________  ________________________________  ________________________________  ________________________________ |  |
| **21** | **D;k bl O;fDr us /;ku ls lquk tc vkius viuh leL;k crkbZ\ D;k vki dgsaxs cgqr vPNs ls] FkksM+k lk ;k fcydqy Hkh ugha\@**Did this person pay attention when you narrated your problem? Would you say very well, somewhat or not at all? | **cgqr vPNs ls@**Very well…………........1  **FkksM+k lk@**Somewhat…………………2  **fcydqy Hkh ugha@**Not at all…………3 |  |
| **22** | **D;k ;g O;fDr vki ij fpYyk;k ;k vkidk etkd cuk;k\@**Did this person shout at you, or make fun of you? | **gka@**Yes…………………………….1  **ugha@**No…………………………....2 | **Q 24** |
| **23** | **bl O;fDr us D;k dgk\@**What did this person say? | ________________________________  ________________________________  ________________________________  ________________________________  ________________________________  ________________________________ |  |
| **24** | **D;k bl O;fDr us vkidks dksbZ loky iwNus dk ekSdk fn;k@ vkidks izsfjr fd;k tks vkidks gks ldrs Fks\@**Did this person give you an opportunity/ encourage you to ask any questions that you may have? | **gka@**Yes…………………………….1  **ugha@**No…………………………....2 |  |
| **25** | **D;k vkius bl O;fDr ls ml leL;k ds ckjs esa crkus ds vykok dksbZ loky iwNsa ftlds fy, vki mtkyk Dyhfud x;s Fks\@**Did you ask this person any questions other than narrating the problem for which you went to the UJALA clinic? | **gka@**Yes…………………………….1  **ugha@**No…………………………....2 | **Q 27** |

| **Q No.** | **Question and Filters** | **Code/Response Categories** | **Skip To** |
| --- | --- | --- | --- |
| **26** | **vkius bl O;fDr ls D;k iwNk\@**What did you ask this person? | ________________________________  ________________________________  ________________________________  ________________________________  ________________________________  ________________________________ |  |
| **27** | **bl O;fDr us vkids lokyksa ds tokc dSls fn;s\@**Howe did this person respond to your questions?  **bl O;fDr ds iwNs x;s lokyksa ds ckjs esa iwNsa( fVIif.k;ka@ voyksdu@ bl O;fDr dh nh xbZ lykg( mlds psgjs ij Hkko½@**Probe about questions this person asked; comments/observations/ advice this person made; his/her facial expressions) | ________________________________  ________________________________  ________________________________  ________________________________  ________________________________  ________________________________ |  |
| **28** | **D;k vki vkidks fn[kk;s x;s lEeku ds laca/k esa mudh izfrfØ;k ds ckjs esa crk;sa] i{kikrh] xSj&i{kikrh joS;k] xksifu;rk cuk;s j[kuk vkfn\ ¼ekSf[kd vkSj xSj&ekSf[kd½@**Can you describe his/her actions in detail in terms of respect shown to you, judgemental/ non-judgemental attitudes, confidentiality maintained ect? (VERBAL AND NON-VERBAL CUES) | ________________________________  ________________________________  ________________________________  ________________________________  ________________________________  ________________________________ |  |
| **29** | **d`i;k tkapsa ;fn DykbaV fdlh ekgokjh leL;k ds fy, xbZ gS@Please check if the client went for a menstrual problem** | |  |
|  | **vki ekgokjh ds nkSjku isV esa rst nnZ vkSj [kwu ds FkDds vkus dh leL;k ds fy, lsok,sa ikus ds fy, Dyhfud xbZ Fkh@**You had gone to the clinic to seek service for severe abdominal pain and clots during periods | |  |
| **a** | **D;k Dyhfud ij vkidks feys fdlh iznkrk us crk;k fd vki bl leL;k dks vuqHko D;ksa dj jgh gks ldrh gSa\ ;fn gka] rks dkSu vkSj mUgksus vkidks D;k crk;k\@**Did any of the providers whom you met at the clinic tell why you may be experiencing this problem? If yes, who and what did they tell you? | _______________________________  _______________________________  _______________________________ |  |

| **Q No.** | **Question and Filters** | **Code/Response Categories** | **Skip To** |
| --- | --- | --- | --- |
| **b** | **D;k Dyhfud ij vkidks feys fdlh iznkrk us lkekU; rkSj ij vkidks ekgokjh ds ckjs esa dqN crk;k\ ;fn gka] rks dkSu vkSj mUgksus vkidks D;k crk;k\@**Did any of the providers whom you met at the clinic tell you anything about periods in general? If yes, who and what did they tell you? | _______________________________  _______________________________  _______________________________ |  |
| **c** | **D;k Dyhfud ij vkidks feys fdlh iznkrk us ekgokjh LoPNrk ds ckjs esa crk;k\ ;fn gka rks dkSu vkSj mUgksus vkidks D;k crk;k\@** Did any of the providers whom you met at the clinic tell about menstrual hygiene? If yes, who and what did they tell you? | _______________________________  _______________________________  _______________________________ |  |
| **d** | **tc vkius oks crk;k tks vkidh nknh us vkidks crk;k] rks mudh izfrfØ;k dSlh Fkh\ D;k Dyhfud ij vkidks feys fdlh iznkrk us vkidh xyr vo/kkj.kk dks Bhd djus dh dksf’k’k dh\ ;fn gka] rks dkSu vkSj mUgksus vkidks D;k crk;k\@**When you mentioned about what your grandmother told you, how did they respond? Did any of the providers whom you met at the clinic try to clear your misconception? If yes, who and what did they tell you? | _______________________________  _______________________________  _______________________________ |  |
| **30** | **D;k Dyhfud ij vkidks feys fdlh iznkrk us vkidh leL;k ds fy, dksbZ mipkj vkidks crk;k\ ;fn gka] rks dkSu\@**Did any of the providers whom you met at the clinic prescribe you any treatment for your problem? If yes, who? | **gka@**Yes…………………………….1  **ugha@**No…………………………....2  **lykg nh@**Gave advice……………..3  **eq>s fdlh vU; iznkrk dks jsQj fd;k@**  Referred me to another provider……4 | Q38 |
| **a** | **;fn gka] rks mUgksus vkidks D;k nok@vkS”kf/k fof/k nh\ D;k mUgksus crk;k fd vkidks nh xbZ nok@vkS”kf/k fof/k dk dSls bLrseky djuk pkfg,\@**If yes, what did they prescribe you? Did they tell you how you should take the prescription?  **bl ckjs esa iwNsa fd nh xbZ nok@vkS”kf/k fof/k dks dSls bLrseky djuk gS ds ckjs esa mUgksus D;k crk;k] iqu% HksaV@**Probe about what they told how to use the prescription, return visits | _______________________________  _______________________________  _______________________________ |  |
| **0b** | **;fn dsoy lykg nh] rks vkidks D;k lykg nh xbZ Fkh\@**If gave advice only, what advice were you given? | _______________________________  _______________________________  _______________________________ |  |

| **Q No.** | **Question and Filters** | **Code/Response Categories** | **Skip To** |
| --- | --- | --- | --- |
| **31** | **;fn DykbaV gLreSFkqu vkSj LoIunks”k ds ckjs esa lykg ysus ds fy, x;k Fkk@**  **If the client has gone for seeking advice about masturbation and nocturnal emission** | |  |
| **a** | **D;k Dyhfud ij vkidks feys fdlh iznkrk us vkidks crk;k fd gLreSFkqu vkSj LoIunks”k D;k gksrk gS\ ;fn gka rks dkSu vkSj mUgksus vkidks D;k crk;k\@**Did any of the providers whom you met at the clinic tell you about what is masturbation and nocturnal emission? If yes, who and what did they tell you? | _______________________________  _______________________________  _______________________________ |  |
| **b** | **tc vkius mUgs crk;k fd vki fpafrr gSa fd vkidks dqN chekjh gS] rks mUgksus dSlh izfrfØ;k nh\ D;k mues ls fdlh us vkidh xyr vo/kkj.kk dks Bhd djus dh dksf’k’k dh\ ;fn gka] rks dkSu vkSj mUgksus vkidks D;k crk;k\@**When you told them that you are worried that you are having some disease, how did they respond? Did any of them try to clear your misconceptions? If yes, who and what did they tell you? |  |  |
| **32** | **;fn DykbaV bl ckjs esa lykg ysus ds fy, xbZ fd vius izseh ds lkFk fj’rs@laca/k dks dSls laHkkyuk gS@**  **If the client has gone to seek advice about how to manage relationship with boyfriend** | |  |
| **a** | **tc vkius iznkrk dks crk;k fd vkidk izseh vkils ;kSu laca/k cukus ds fy, ncko Mky jgk gS vkSj vkidks irk ugha fd bls dSls laHkkyuk gS rks mUgksus dSlh izfrfØ;k nh\ D;k mUgksus bl ckjs esa lykg nh fd bl fLFkfr dks dSls laHkkyuk gS\ ;fn gka rks dkSu vkSj mUgksus vkidks D;k crk;k\@**When you told the provider that your boyfriend is pressurizing you to engage in sex and you don’t know how to handle, how did they respond? Did they advise you about how to handle the situation? If yes, who and what did they tell you? | _______________________________  _______________________________  _______________________________ |  |
| **b** | **vkius ftuls ckr fd D;k mues ls fdlh us vkidks crk;k fd xHkZorh gksus ls dSls cpk tk ldrk gS\ ;fn gka rks dkSu vkSj mUgksus D;k dgk\@**Did any of them talk to you about how you may avoid getting pregnant? If yes, who and what did they say? | _______________________________  _______________________________  _______________________________ |  |
| **c** | **vkius ftuls ckr dh D;k mues ls fdlh us vkids ugha dgus ds vkids vf/kdkj ds ckjs esa crk;k\ ;fn gka] rks dkSu vkSj mUgksus D;k dgk\@**Did any of them talk to you about your right to say no? If yes, who and what did they say? | _______________________________  _______________________________  _______________________________ |  |

| **Q No.** | **Question and Filters** | **Code/Response Categories** | | **Skip To** |
| --- | --- | --- | --- | --- |
| **33** | **;fn DykbaV vupkgh xHkkZoLFkk ls fuiVus ds fy, tkudkjh ysus xbZ@**  **If the client has gone for information for managing unwanted pregnancy** | | |  |
| **a** | **tc vkius ekgokjh uk gksus vkSj detksjh okyh viuh leL;k ds ckjs esa crk;k] rks mUgksus dSlh izfrfØ;k nh\@**When you narrated your problem of missed period and weakness, how did they respond? What questions did they ask you and who was this person? | _______________________________  _______________________________  _______________________________ | |  |
| **b** | **D;k mues ls fdlh us vkidks xHkkZoLFkk tkap fdV ds ckjs esa crk;k\ ;fn gka] rks dkSu vkSj mUgksus vkidks D;k crk;k\@**Did any of them tell you about pregnancy test kit? If yes, who and what did they tell you? | _______________________________  _______________________________  _______________________________ | |  |
| **c** | **D;k mues ls fdlh us vkidks xHkZikr ds ckjs esa crk;k\ ;fn gka] rks dkSu vkSj mUgksus vkidks D;k crk;k\@**Did any of them tell you about abortion? If yes, who and what did they tell you? | _______________________________  _______________________________  _______________________________ | |  |
| **d** | **D;k mues ls fdlh us vkidks xHkZfujks/kd ds ckjs esa crk;k\ ;fn gka] rks dkSu vkSj mUgksus vkidks D;k crk;k\ dgka ls ikuk gS\ dSls bLrseky djuk gS\@**Did any of them tell you about contraception? If yes, who and what did they tell you? Where to get? How to use? | _______________________________  _______________________________  _______________________________ | |  |
| **e** | **D;k mUgksus vkids ;kSu vuqHkoksa ds ckjs esa iwNk\ ;fn gka] rks dkSu vkSj mUgksus vkils D;k iwNk\@**Did they ask about your sexual experiences? If yes, who and what did they ask? | _______________________________  _______________________________  _______________________________ | |  |
| **f** | **d`i;k tkpsa ;fn] DykbaV ,lVhvkbZ vkSj xHkZfujks/kd tkudkjh ds fy, vk;k gS**  **PLEASE CHECK IF, the client came for STI and Contraceptive information** | **gka@**  **YES** | **iz’u 34 ij tk;sa@**  **Go to Question 34** |  |
|  |  | **ugha@**  **NO** | **iz’u 40 ij tk;sa@**  **Go to Question 40** |  |
| 34 | **D;k fdlh iznkrk us vkids ;kSu bfrgkl ds ckjs esa tkudkjh yh\ ;fn gka rks dkSu\@**Did any of the providers take your sexual history? If yes, who? | _______________________________  _______________________________  _______________________________ | |  |
| 35 | **D;k mues ls fdlh us vkidks xHkZfujks/kd ds ckjs esa crk;k\ ;fn gka] rks dkSu vkSj mUgksus vkidks D;k crk;k\ dgka ls ikuk gS\ dSls bLrseky djuk gS\@**Did any of them tell you about contraceptives? If yes, who and what did they tell you? What you should use, how you should use, where you can get? | _______________________________  _______________________________  _______________________________ | |  |
| 36 | **D;k mues ls fdlh iznkrk us vkidks dksbZ tkap djkus ds fy, dgk\ ;fn gka] rks dkSu\ dkSu lh tkap\ dgka\@**Did any of the provider tell you about going for any test? If yes, who? What tests? Where? | _______________________________  _______________________________  _______________________________ | |  |

| **Q No.** | **Question and Filters** | **Code/Response Categories** | **Skip To** |
| --- | --- | --- | --- |
| 37 | **D;k mues ls fdlh iznkrk us vkidks vius lkFkh dh dksbZ tkap djkus ds fy, dgk\ ;fn gka] rks dkSu\ dkSu lh tkap\ dgka\@**Did any of the provider tell you about partner getting tested? If yes, who, what tests? Where? | _______________________________  _______________________________  _______________________________ |  |
| 38 | **D;k mues ls fdlh us vkils ,lVhvkbZ ds ckjs esa ckr dh\ ;fn gka rks dkSu\ mUgksus vkidks D;k crk;k\@**Did any of them to talk to you about STIs? If yes, who? What did they tell you? | **gka@**Yes…………………………….1  **ugha@**No…………………………....2 | Q40 |
| 39 | **D;k mues ls fdlh us vkils ,pvkbZoh@ ,M~l ds ckjs esa ckr dh\ ;fn gka] rks dkSu vkSj mUgksus vkidks D;k crk;k\@**Did any of them talk to you about HIV/AIDS? If yes, who and what did they tell you? | _______________________________  _______________________________  _______________________________ |  |
|  | **lHkh ds fy,@FOR ALL** |  |  |
| 40 | **D;k iznkrkvksa us vkidks bl rjhds ls crk;k fd vkidks vklkuh ls le> vk tk;s\ ;fn gka rks dkSu\ ;fn ugha rks dkSu\@**Did the providers you met explain the things in a way that you understood easily? If yes, who? If no, who | _______________________________  _______________________________  _______________________________ |  |
| 41 | **;fn ugha] rks ckrphr le>us esa vkius dkSu lh pqukSfr;ksa dk lkeuk fd;k\**  If not, what challenges did you face in understanding the conversation? | _______________________________  _______________________________  _______________________________ |  |
| 42 | **D;k leL;k ds ckjs esa T;knk i<+us ds fy, vkidks dksbZ ipkZ feyk\ ;fn gka] rks d`i;k mldh izfr ysa@**Did you get a handout/brochure to read more about the problem? If yes, please take the copy | **gka@**Yes…………………………….1  **ugha@**No…………………………....2 |  |
| 43 | **D;k iznkrk us vkidks Hkjkslk fnyk;k fd ;fn vko’;d gks rks vki nksckjk vk ldrs gSa\ ;fn gka rks dkSu\@**Did the provider reassure you that if necessary you could return? If yes, who? | _______________________________  _______________________________  _______________________________ |  |
| 44 | **D;k iznkrk us vkils ekrk&firk dh vuqefr@lgefr ykus ds fy, dgk\ ;fn gka rks dkSu\@**Did the provider require you to get parental consent? If yes, who? | **gka@**Yes…………………………….1  **ugha@**No…………………………....2 |  |
| 45 | **dqyfeykdj] vki lqfo/kk dsUnz ij vkils fd;s x;s O;ogkj ls larq”V Fks\@**Overall, were you satisfied with the way you were treated at the facility? | **gka@**Yes…………………………….1  **ugha@**No…………………………....2 |  |
| 46 | **D;k vkidks yxrk gS dksbZ ;qok O;fDr ftls okLro esa leL;k gks ftlds fy, vki enn [kkst jgs Fks D;k mls bl dsUnz ls vPNh lsok feysxh\ ;fn ugha] rks D;k djus dh tjwjr gS\** Do you think a young person who really has the problem you were seeking help for would get good service from this centre? If not, what needs to be done? | _______________________________  _______________________________  _______________________________ |  |

| **Q No.** | **Question and Filters** | **Code/Response Categories** | **Skip To** |
| --- | --- | --- | --- |
| **voyksdu@OBSERVATIONS** | | |  |
| **47** | **D;k lqfo/kk dsUnz LoPN Fkk\**  Was the facility clean? | **gka@**Yes…………………………….1  **ugha@**No……………………………..2 |  |
| **48** | **D;k DykbaV~l ds fy, pkyw voLFkk esa vyx&vyx ‘kkSpky; Fks\**  Were there separate functional toilets for clients? | **gka@**Yes…………………………….1  **ugha@**No……………………………..2 |  |
| **49** | **D;k lqfo/kk dsUnz ij ihus ds ikuh dh lqfo/kk Fkh\**  Was drinking water available at the facility? | **gka@**Yes…………………………….1  **ugha@**No……………………………..2 |  |
