# Supplementary material for: Assessment of the quality of sexual and reproductive health services delivered to adolescents at Ujala clinics: A qualitative study in Rajasthan, India
Source: PLoS One. 2022 Jan 10;17(1):e0261757. doi: 10.1371/journal.pone.0261757 (PMC8746710; doi:10.1371/journal.pone.0261757)
Supplement: S4 Appendix — (DOCX) [file pone.0261757.s004.docx]

**S4 Appendix: In-Depth Interview Guide for Counsellors**

| **State**  **राज्‍य** |  |
| --- | --- |
| **District code**  जिला कोड |  |
| **Block code**  ब्‍लॉक कोड |  |
| **Clinic code**  क्‍लीनिक कोड |  |
| **Village code**  **गांव कोड** |  |
| **Sex of the respondent**  **प्रतिभागी पुरूष है या महिला** | **Male Female**  **पुरूष महिला** |
| **Audio-recoding of IDI permitted**  **आईडीआई की ऑडियो-रिकार्डिंग की अनुमति दी गई** | **Yes**  **हां NO नहीं** |
| **Date the IDI was conducted**  **आईडीआई करने की तारीख** | **____ ___ _____**  **MM / DD /YYYY** |
| **Name of the person who conducted the IDI**  आईडीआई करने वाले व्‍यक्ति का नाम | **______________________________** |

**Background पृष्‍ठभूमि**

“Namaskar, my name is __________. I am working with Population Council. We are conducting a study to learn about health concerns of adolescents in your district, health services available to them, the extent to which adolescents access these services and how these services can be made more accessible to adolescents. We are also interested in learning about the experiences of counsellors in providing services to adolescents and the constraints that they face in doing so. We want to interview some counsellors who have been providing counselling services at adolescent health ‘Ujala clinics’ and would be knowledgable about the lives and needs of adolescents. Your ideas and opinions will help us to make recommendations to the health department for implementing health services that are responsive to adolescents and we value your participation.

“नमस्‍कार, मेरा नाम .................. है। मैं पॉपुलेशन कौंसिल के साथ काम कर रहा हूं। हम आपके जिले में किशोरों/ किशोरियों की स्‍वास्‍थ्‍य समस्‍याओं, उनके लिए उपलब्‍ध सेवाओं, किशोर/ किशोरी इन सेवाओं को किस हद तक इस्‍तेमाल करते हैं और ये सेवाऐं किशोरों/ किशोरियों को कैसे ज्‍यादा से ज्‍यादा उपलब्‍ध कराई जा सकती हैं, उसके बारे में सीखने के लिए एक सर्वे कर रहे हैं। किशोरों को ये सेवाऐं प्रदान करने में काउंसलर्स के अनुभवों और ऐसा करने में उनके सामने आने वाली रूकावटों के बारे में सीखने में हम इच्‍छुक हैं। हम कुछ काउंसलर्स के साथ इंटरव्‍यू करना चाहते हैं जो किशोर/ किशोरी को स्‍वास्‍थ्‍य ‘उजाला क्‍लीनिक्‍स’ पर सलाह सेवाऐं प्रदान कर रहे हैं और जो किशोरों/ किशोरियों की जिंदगी और जरूरतों के बारे में जानकारी रखते हैं। आपके विचारों और राय से हमे स्‍वास्‍थ्‍य विभाग को स्‍वास्‍थ्‍य सेवाऐं लागू करने के लिए सुझाव/ सिफारिश करने में मदद मिलेगी जो किशोरों/ किशोरियों की जरूरतें पूरी करती हैं और हम आपकी भागीदारी की कद्र करते हैं।

As we told you earlier also, your participation is completely voluntary. You may end the participation in the IDI at any time. Your name or other information that could identify you will not appear on the study record or report.

जैसा हमने आपको पहले भी बताया था, आपकी भागीदारी पूरी तरह से स्‍वैच्छिक हैं आप किसी भी समय आईडीआई में भागीदारी समाप्‍त कर सकते हैं। आपका नाम या अन्‍य जानकारी जिनसे आपकी पहचान हो सकती है उसे अध्‍ययन में रिकार्ड या रिपोर्ट में नजर नहीं आयेंगे।

Thanks again for letting me interview you today. Please remember that everything you tell me will be confidential and I will not write down your name anywhere. I would like to record our conversation so that I don’t miss any important information given by you. Please note that this would be used solely for the research purpose and will not be shared with anyone else.

मुझे आपका इंटरव्‍यू करने देने के लिए धन्‍यवाद। कृपया याद रखें कि जो कुछ भी आप मुझे बताते हैं वह गोपनीय रहेगा और मैं आपका नाम कहीं पर भी नहीं लिखूंगा। मैं हमारी इस बातचीत को रिकार्ड करना चाहूंगा ताकि आपके द्वारा दी जाने वाली कोई महत्‍वपूर्ण जानकारी मुझसे छूट ना जाये। कृपया ध्‍यान रखें कि यह पूरी तरह से केवल रिसर्च उद्देश्‍य के लिए है और किसी के साथ साझा नहीं किया जायेगा।

May I please use the tape to record this conversation? (If use of tape recorder was approved), let me turn on the tape recorder and we can begin. If not, then continue the discussion and add detailed notes wherever possible.

क्‍या मैं इस बातचीत को रिकार्ड करने के लिए टेप इस्‍तेमाल कर सकता हूं? (यदि टेप रिकार्डर के इस्‍तेमाल की अनुमति दी गई), मुझे टेप रिकार्डर ऑन करने दें और उसके बाद बस हम शुरू कर सकते हैं। यदि नहीं, तो बातचीत को जारी रखें और जहां कहीं भी संभव हो विस्‍तृत नोट दर्ज करें।

1. Current role – understanding of role, likes and dislikes about the current role, knowledge, supervisor support, system support, average time spent on counseling, other roles and responsibilities, challenges, need and support required.

वर्तमान भूमिका - भूमिका, वर्तमान भूमिका के बारे में पसंद और नापसंद, भूमिका के बारे में जानकारी, सुपरवाइजर सहयोग, सिस्‍टम सहयोग, सलाह देने में बिताया गया औसत समय, अन्‍य भूमिकाऐं और जिम्‍मेदारियां, चुनौतियां, जरूरत और आवश्‍यक सहयोग को समझना।

1. Understanding of adolescents’ issues – perception about adolescents, their special needs,

किशोर/ किशोरी की समस्‍याओं को समझना - किशोरों/ किशोरियों के बारे में समझ/ धारणा, उनकी विशेष जरूरतें,

**A. WARM UP** शुरूआत करना

**A1: Background** पृष्‍ठभूमि

1. Please tell me about yourself?

कृपया मुझे अपने बारे में बतायें?

- 1. What is your age? Education background? Where do you live? How far is your village from the facility? do you do other jobs (apart from counsellor) as well?

आपकी उम्र क्‍या है? शैक्षिक पृष्‍ठभूमि? आप कहां रहते हैं? आपका गांव सुविधा केन्‍द्र से कितनी दूर है? क्‍या आप अन्‍य काम भी करते हैं (काउंसलर के अलावा)?

1. What about your family? When did you get married? Children? Others staying with you?

अपने परिवार के बारे में बतायें? आपका विवाह कब हुआ था? बच्‍चे? आपके साथ रहने वाले अन्‍य लोग?

1. When do you usually have free time – time for yourself? What do you do in your free time? What are your dreams and aspirations for yourself? Your children? How are you working towards achieving these goals? PROBE TO UNDERSTAND THE SOCIAL PROFILE OF THIS COUNSELLOR

आमतौर पर आपके पास खाली है समय कब होता – जैसे की अपने लिए समय कब होता है? आप अपने खाली समय में क्या करते हैं? आपके खुद के लिए आपके क्‍या सपने और आकांक्षाऐं हैं? आपके बच्‍चे? अपने इन लक्ष्‍यों को पाने के संबंध में आप कैसे काम कर रहे हैं? इस काउंसलर की सामाजिक रूपरेखा समझने के लिए प्रोब करें

**B. EXPERIENCE OF WORKING AS A COUNSELLOR**

काउंसलर के तौर पर काम करने का अनुभव

**B1: Tell me about your experience of working as a counsellor?**

मुझे काउंसलर के तौर पर काम करने के अपने अनुभव के बारे में बतायें?

1. What made you interested in the counsellor position? Did you apply or you were nominated by someone else? Have you worked anywhere before this job? What were the positions in which you have worked? LIST DOWN THE POSITION DETAILS.

आप किससे प्रेरित होकर काउंलसर पोजीशन में इच्‍छुक हुए? आपने क्‍या आवेदन किया था या आप किसी के द्वारा नामांकित किये गये थे? क्‍या आपने इस नौकरी से पहले कहीं और काम किया है? आपने वहां किस पद पर काम किया था ? पद विवरण की सूची बनायें

1. How long you have been working as counsellor? At this facility? Were you placed at some other Government facility before?

आप काउंसलर के तौर पर कितने समय से काम कर रहे हैं? इस सुविधा केंद्र पर? क्‍या आप पहले किसी अन्‍य सरकारी सुविधा केंद्र पर काम कर रहे थे?

1. Did you attend some formal course to become a counselor? Were they part of your studies or trainings imparted on the job? LIST DOWN THE TRAINING/COURSES ATTENDED.

क्‍या आपने काउंसलर बनने से पहले कोई औपचारिक कोर्स किया था? क्‍या कोर्स आपकी पढ़ाई का हिस्सा था या नौकरी में काम करने के तौर पर प्रशिक्षण का हिस्सा था ? भाग लिये गये प्रशिक्षण/ कोर्सेज की सूची बनायें।

**B2: Working in UjalaClinic**

**उजाला क्‍लीनिक में काम करना**

1. When did you start working in adolescent friendly health clinic? What made you interested in this position? Did you apply or you were nominated? Were you there from the start of Ujalaclinic? Was there someone else who counseled the adolescents before/ worked along with you?

आपने किशोरों/ किशोरियों के स्‍वास्‍थ्‍य क्‍लीनिक में कब काम करना शुरू किया था? आप किससे प्रेरित होकर इस पद में इच्‍छुक हुए? क्‍या आपने आवेदन किया था या आप नामांकित किये गये थे? उजाला क्‍लीनिक शुरू होने के समय से ही क्‍या आप यहां हैं ? क्‍या पहले कोई अन्‍य था जिन्‍होने किशोरों/ किशोरियों को सलाह दी थी या आपके साथ किशोरों/ किशोरियों को सलाह दी थी?

1. Have you ever attended any training programme focusing on adolescent health? ***If yes***, can you tell me more about that training programme? When did you attend it? How many days long was the training progamme? What were the topics covered in the training programme? What did you learn? Could you tell me some of the new things that you learned in the area of adolescent health? Did you find that training useful? Why or why not?

क्‍या आपने कभी किशोरों/ किशोरियों के स्‍वास्‍थ्‍य पर केन्द्रित किसी प्रशिक्षण कार्यक्रम में भाग लिया है? यदि हां, क्‍या आप मुझे उस प्रशिक्षण कार्यक्रम के बारे में और कुछ बता सकते हैं? आपने इसमे कब भाग लिया था? प्रशिक्षण कार्यक्रम कितने दिनों तक चला था? प्रशिक्षण कार्यक्रम में कौन से विषय कवर किये गये थे? आपने क्‍या सीखा था? क्‍या आप मुझे कुछ नई चीजें बता सकते हैं जो आपने किशोरों/ किशोरियों के स्‍वास्‍थ्‍य के बारे में सीखी? क्‍या आपको वह प्रशिक्षण उपयोगी लगा? क्‍यों या क्‍यों नहीं?

1. ***If no,*** in any of the training programmes that you have attended, was the topic of adolescent health discussed? What did you learn? Could you tell me some of the new things that you learned in the area of adolescent health?

***यदि नहीं, आपके भाग लिये गये किसी भी प्रशिक्षण कार्यक्रम में, क्‍या*** किशोरों/ किशोरियों के स्‍वास्‍थ्‍य के विषय पर चर्चा की गई थी? आपने क्‍या सीखा? क्‍या आप मुझे कुछ नई चीजें बता सकते हैं जो आपने किशोरों/ किशोरियों के स्‍वास्‍थ्‍य के बारे में सीखी?

1. Did you receive any training/orientation for this role? Could you please describe the details – how long? Who undertook the training? State level, regional or district? Were you oriented to counsel adolescent in a specific way? Can you remember what were you told? Do you think that is relevant? Do you think people usually practice them? Why and why not?

क्‍या आपको इस भूमिका के लिए कोई प्रशिक्षण/ ओरियंटेशन प्राप्‍त हुआ था? कृपया आप विस्‍तार में बतायें - कितना लंबा प्रशिक्षण/ ओरियंटेशन? प्रशिक्षण किसने दिया था? राज्‍य स्‍तर, क्षेत्रीय या जिला? क्‍या आपको किशोरों/ किशोरियों को किसी विशेष तरीके से सलाह देने के लिए सिखाया गया था? क्‍या आप याद कर सकते हैं कि आपको क्‍या बताया गया था? क्‍या आपको लगता है यह उपयुक्‍त है? क्‍या आपको लगता है लोग आमतौर पर उनका अभ्‍यास करते हैं? क्‍यों और क्‍यों नहीं?

1. How long have you been working in this clinic? What is the nature of job that you handle? Are there other tasks (apart from counseling) assigned to you? How frequently do you come to the facility? For how long do you stay at the facility?

आप इस क्‍लीनिक में कितने समय से काम कर रहे है? आप यहां किस प्रकार का काम संभालते हैं? क्‍या आपको कोई अन्‍य काम भी दिये गये हैं (सलाह देने के अलावा)? आप सुविधा पर कब-कब आते हैं? आप सुविधा पर कितने समय के लिए ठहरते हैं?

1. How much time do you spend in counseling adolescents? Who supervises your work? Whom do you reach out in case of any queries?

आप किशोरों/ किशोरियों को सलाह देने में कितना समय बिताते हैं? आपके काम को कौन सूपर्वाइज़ करता है? किसी समस्‍या के होने पर आप किससे संपर्क करते हैं?

1. How do you manage your family responsibility along with this job? Do you face any difficulties in managing this work? What do you like and dislike about this job?

आप इस काम के साथ में अपनी पारिवारिक जिम्‍मेदारियों को कैसे संभालते हैं? क्‍या आप इस काम को करने में किसी मुश्किल का सामना करते हैं? आपको इस काम के बारे में क्‍या पसंद और नापसंद है?

**C. DEALING WITH UNMARRIED ADOLESCENTS AND THEIR HEALTH ISSUES**

अविवाहित किशोरों/ किशोरियों और उनकी स्‍वास्‍थ्‍य समस्‍याओं से निपटना

**C1. Adolescent’s Client Profile**

किशोरों/ किशोरियों की क्‍लाइंट रूपरेखा

1. At the facility in which you are currently working, which category of adolescents does typically seek services? By category of adolscents girls and boys, we mean, younger or older adolescents, married or un-married adolescents, school or out of school adolescents, working or non-working. **PROBE TO GET A PROFILE OF THE CLIENTS (male and female) VISITING THE CLINIC.**

जिस सुविधा पर आप आजकल काम कर रहे हैं वहां, आमतौर पर किस श्रेणी के किशोर/ किशोरियां सेवाऐं लेने के लिए आते हैं? किशोरों/ किशोरियों की श्रेणी से, हमारा मतलब, युवा या बड़े किशोर/ किशोरियां, विवाहित या अविवाहित किशोर/ किशोरियां, स्‍कूल जाने वाले या स्‍कूल नहीं जाने वाले किशोर/ किशोरियां, कामकाजी या काम नहीं करने वाले किशोर/ किशोरियां। **क्‍लीनिक पर आने वाले लोगों की रूपरेखा (पुरूष और महिला) जानने के लिए प्रोब करें।**

LET’S FOCUS ON UNMARRIED ADOLESCENTS FOR THE FOLLOWING DISCUSSION

आगे की चर्चा के लिए आइये अब हम अविवाहित किशोरों/ किशोरियों पर ध्‍यान केन्द्रित करते हैं

1. How many adolescents come to the UjalaARSH clinic in a week? Focusing on UNMARRIED ADOLESCENTS, do you think this number is high or low? Why do you think so? Are there other providers where these adolescents go to seek counseling and information?

एक हफ्‍ते में कितने किशोर/ किशोरियां उजाला अर्श क्‍लीनिक में आते हैं? अविवाहित किशोरों/ किशोरियों पर ध्‍यान केन्द्रित करते हुए, क्‍या आपको लगता है यह नंबर अधिक या कम है? आपको ऐसा क्‍यों लगता हे? क्‍या कोई अन्‍य प्रदाता हैं जहां ये किशोर/ किशोरियां सलाह और जानकारी लेने के लिए जाते हैं?

1. Why do you think adolescents do not access these services? (Probe about lack of awareness about the clinic among adolescents?? Inconvenient timing of the clinic?? Concerns among adolescents about privacy, confidentiality etc?? HCP not being able to give time/attention to the adolescents?? Lack of family support??) What might help to overcome these barriers?

आपके अनुसार किशोर/ किशोरियां ये सेवाऐं क्‍यों इस्‍तेमाल नहीं करते हैं? (किशोर/ किशोरियों के बीच क्‍लीनिक के बारे में जागरूकता की कमी के बारे में प्रोब करें?? क्‍लीनिक का असुविधाजनक समय?? किशोर/ किशोरियों के बीच निजता, गोपनियता आदि के बारे में चिंताऐं?? स्‍वास्‍थ्‍य प्रदाता (एचसीपी) किशोर/ किशोरियों को समय/ ध्‍यान दे पाने में सक्षम नहीं? पारिवारिक सहयोग की कमी??) इन रूकावटों को दूर करने में क्या मदद मिल सकती है?

1. Do you try other strategies/ways (apart from those coming to your clinic by themselves) to cater to higher number of adolescents? **CHECK** how he/she use different ways to reach out to male and female adolescents.

क्‍या आप अधिक संख्‍या में किशोर/ किशोरियों की जरूरतों को पूरा करने के लिए अन्‍य नीतियां/ तरीके (उनके अलाव जो अपने आप से आपके क्‍लीनिक में आते हें) कोशिश करते हैं? जांचें वह किशोर/ किशोरियों तक पहुंचने के लिए कैसे विभिन्‍न तरीके इस्‍तेमाल करते हैं?

1. In case, there are no unmarried adolescents coming to clinic ask the counsellor about what they think are the reasons of why adolescents are not coming to the clinic?

यदि, कोई अविवाहित किशोर/ किशोरियां क्‍लीनिक नहीं आ रहे हैं तो काउंसलर से पूछें उनके अनुसार क्‍या कारण हैं कि किशोर/ किशोरियां क्‍लीनिक पर नहीं आ रहे हैं?

**C2. Adolescent’s health issues**

**किशोर/ किशोरियों की स्‍वास्‍थ्‍य समस्‍याऐं**

Talking specifically about the UNMARRIED adolescents (15-19 years)

खासतौर पर अविवाहित किशोर/ किशोरियों (15-19 साल उम्र) के बारे में बात करते हुए

1. What kind of health issues do adolescent girls and boys seek counselling on? How many average number of adolescents visit this clinic in a week? With what kind of health issues? In case of no clients’ footfall, ask them about what they think are the health issues on which adolescent girls and boys in the community need further information and counselling?

किशोर/ किशोरियां किस प्रकार की समस्‍याओं पर सलाह लेने आते हैं? एक हफ्‍ते में औसतन कितनी संख्‍या में किशोर/ किशोरियां क्‍लीनिक पर आते हैं? यदि कोई किशोर/ किशोरियां नहीं आते हैं, तो उनसे पूछें उनके अनुसार वे कौन सी समस्‍याऐं हैं जिन पर समुदाय में किशोर/ किशोरियों को और जानकारी और सलाह की जरूरत है?

1. In your opinion, where are the adolescents seeking SRH information from? Do you think those are reliable sources? What kind of information is available to them?

आपकी राय में, किशोर/ किशोरियां एसआरएच जानकारी कहां से ले रहे हैं? क्‍या आपको लगता है वे विश्‍वसनीय स्‍त्रोत हैं? उनके पास किस प्रकार की जानकारी उपलब्‍ध है?

1. Do you think adolescent needs special clinics (Ujala clinics) to access SRH related information? Why, please explain. Can’t they be treated like everyone else (probe that isn’t it the same doctor who treats other clients as well, then why?)? Do they feel comfortable talking to a health provider? To you?

क्‍या आपको लगता है किशोर/ किशोरियों को एसआरएच संबंधित जानकारी इस्‍तेमाल करने के लिए विशेष क्‍लीनिक्‍स (उजाला क्‍लीनिक्‍स) की जरूरत है? क्‍यों, कृपया समझायें। क्‍या उनका हर किसी के समान व्यवहार नहीं किया जा सकता है (प्रोब करें कि क्‍या यह वही डॉक्टर नहीं है जो दूसरों का भी उपचार करते हैं, तो क्‍यों)? क्‍या उन्‍हे स्‍वास्‍थ्‍य प्रदाता से बात करना सहज लगता है? आपसे बात करना?

**D. ROLE OF ARSH COUNSELLOR**

**अर्श काउंसलर की भूमिका**

**D1. Counselling adolescents on SRH issues**

**किशोर/ किशोरियों को एसआरएच समस्‍याओं पर सलाह देना**

1. What role can counsellor play in providing SRH information to adolescents? How easy/difficult it would be for counsellors to do so? Why so? What kind of support do counsellors need to provide SRH information to adolescents?

काउंसलर किशोर/ किशोरियों को एसआरएच जानकारी प्रदान करने में क्‍या भूमिका निभा सकते हैं? काउंसलर्स के लिए ऐसा करना कितना आसान/ मुश्किल होगा? ऐसा क्‍यों? काउंसलर्स को किशोर/ किशोरियों को एसआरएच जानकारी प्रदान करने में किस प्रकार के सहयोग की जरूरत है?

1. How do you treat the adolescents who come to your clinic for counselling services? Are there some specific rules that you follow while dealing with them? What are those rules? Why do you think they are important? (if no clients, then ask them to imagine about how they would treat if an adolescent comes to their clinic)

आप किशोर/ किशोरियों से कैसा व्‍यवहार करते हैं जो सलाहसेवाओं के लिए आपके क्‍लीनिक पर आते हैं? क्‍या कोई विशेष नियम हैं जिनका आप उनसे बातचीत करते समय पालन करते हैं? वे नियम क्‍या हैं? आपके अनुसार वे महत्‍वपूर्ण क्‍यों हैं? (यदि वहां कोई मौजूद नहीं है, तो उनसे कल्‍पना करने के लिए कहें कि वे कैसा व्‍यवहार करेंगे यदि कोई किशोर/ किशोरी उनके क्‍लीनिक में आते हैं)

1. Do you use any support material (flipchart/brochure/leaflets) during your counselling session? Where did you get these materials from? Do you think they are effective? Do you give anything to adolescents for take-away?

क्‍या आप अपने सलाह सत्रों के दौरान कोई सहयोगी सामग्री (फ्लिपचार्ट/ ब्रोशर/ लीफलेट्स) इस्‍तेमाल करते हैं? आपको यह सामग्रियां कहां से मिली? क्‍या आपको लगता है वे असरदार हैं? क्‍या आप किशोर/ किशोरियों को साथ ले जाने के लिए कुछ देते हैं?

1. In your view, how equipped is the clinic for addressing the needs of adolescents (UNMARRIED)? [probe about ensuring privacy, confidentiality, non-judgmental services, continuum of care, availability of providers, adequate time to listen to the adolescents, availability of informational materials, adequate supply of medicines, contraceptives etc?]

आपके विचार में, क्‍लीनिक किशोर/ किशोरियों (अविवाहित) की जरूरतों को पूरा करने के लिए कितना लैस/ सक्षम है? (निजता, गोपनियता सुनिश्चित करना, गैर-आलोचनात्‍मक सेवाऐं, देखभाल जारी रखना, प्रदाताओं की उपलब्‍धता, किशोर/ किशोरियों को सुनने के लिए पर्याप्‍त समय, जानकारीपूर्ण सामग्रियों की उपलबधता, दवाओं की पर्याप्‍त आपूर्ति, गर्भनिरोधकों आदि के बारे में प्रोब करें?)

1. Do you feel confident of handling adolescent health related queries independently? Queries related to boys and girls, issues related to Mensruation, nightfall, genital infections, sexual relationships, contraceptives and abortion services etc. Do you feel comfortable in talking to unmarried adolescents about these issues? Has someone ever come to you for this information? Can you share some example? What did you do?

क्‍या आप किशोर/ किशोरियों की स्‍वास्‍थ्‍य संबंधित समस्‍याओं को अकेले संभालने में आश्‍वस्‍त महसूस करते हैं? लड़कों और लड़कियों से संबंधित शंकाऐं, माहवारी, स्‍वप्‍नदोष, यौन संक्रमण, यौन संबंध, गर्भनिरोधकों और गर्भपात सेवाओं आदि से संबंधित समस्‍याऐं। क्‍या आपको अविवाहित किशोर/ किशोरियों से इन मसलों के बारे में बात करना सहज लगता है? क्‍या कभी कोई आपके पास इस जानकारी के लिए आये हैं? क्‍या आप कुछ उदाहरण दे सकते हैं? आपने क्‍या किया था?

1. Did you ever feel lack of information on any topic? Whom do you reach out for support? Did you get the required information? Do you feel capable of addressing that query in case it comes again?

क्‍या आपको कभी किसी विषय पर जानकारी की कमी महसूस हुई हे? आप सहयोग लेने के लिए किसके पास जाते हैं? क्‍या आपको आवश्‍यक जानकारी मिली? क्‍या आप उस समस्‍या को हल करने में सक्षम महसूस करते हैं यदि दोबारा होती है?

1. In case of no previous clients, ask them as how would they counsel an unmarried girl/an unmarried boy who has approached them to seek counseling for problems related to physical mastubation?

पिछले कोई क्‍लाइंट ना होने की स्थिति में, उनसे पूछें वे किसी अविवाहित लड़की/ अविवाहित लड़के को कैसे परामर्श करेंगे जिसने उनसे शारीरिक हस्‍तमैथुन से संबंधित समस्‍याओं के लिए सलाहलेने के लिए उनसे संपर्क किया?

1. In your opinion, when should adolescent girls receive information on SRH matters – before they attain puberty or after, before marriage or after? Why so? What are some important topics that adolescent girls should know about? Who should provide this information to them? Why so?

आपकी राय में, किशोरी लड़कियों को एसआरएच मसलों पर जानकारी कब प्राप्‍त करनी चाहिए – यौन अवस्था शुरू से पहले या बाद में, विवाह से पहले या बाद में? ऐसा क्‍यों? वे कौन से कुछ महत्‍वपूर्ण विषय हैं जिनके बारे में किसी किशोरी लड़की को जानना चाहिए? यह जानकारी उन्‍हे किसके द्वारा प्रदान की जानी चाहिए? ऐसा क्‍यों?

1. In your opinion, when should adolescent boys receive information on SRH matters – before they attain puberty or after, before marriage or after? Why so? What are some important topics that adolescent boys should know about? Who should provide this information to them? Why so?

आपकी राय में, किशोर लड़कों को एसआरएच मसलों पर जानकारी कब प्राप्‍त करनी चाहिए - यौन अवस्था शुरू से पहले या बाद में, विवाह से पहले या बाद में? ऐसा क्‍यों? वे कौन से कुछ महत्‍वपूर्ण विषय हैं जिनके बारे में किसी किशोर लड़के को जानना चाहिए? यह जानकारी उन्‍हे किसके द्वारा प्रदान की जानी चाहिए? ऐसा क्‍यों?

1. In your opinion, what are the ways of making adoelscents aware about the Ujala clinics? Do you make school visits to talk to adolescents? Do you use any IEC material? What are these materials? Any other way?

आपकी राय में, वे किशोर/ किशोरियों को उजाला क्‍लीनिक्‍स के बारे में जानकारी देने के कौन से तरीके हैं? क्‍या आप किशोर/ किशोरियों से बात करने के लिए स्‍कूल में जाते हैं? क्‍या आप किसी आईईसी सामग्रियों का इस्‍तेमाल करते हैं? ये सामग्रियां क्‍या हैं? कोई अन्‍य तरीका?

In your views, what could be the reasons that could restrict adolescents to avail the serives at the facility?

आपके विचार में, क्‍या कारण हो सकते हैं जो किशोर/ किशोरियों को सुविधा में सेवाऐं लेने से रोक सकते हैं?

**D2. Health services and referrals**

स्‍वास्‍थ्‍य सेवाऐं और रेफरल्‍स

1. In your opinion, what type of health services do adolescents in your community require? Who should provide these services? [probe specifically about ASHA, ANM, MO, private practitioners in the village?] At which facilities should these services be made available to adolescents? [probe specifically about community level, sub-centre, PHC and CHC]

आपकी राय में, आपके समुदाय में किशोर/ किशोरियों को किस प्रकार की स्‍वास्‍थ्‍य सेवाओं की आवश्‍यकता है? ये सेवाऐं किसे प्रदान करनी चाहिऐं? (खासतौर पर गांव में आशा, एएनएम, एमओ, निजी डॉक्‍टर के बारे में प्रोब करें?) ये सेवाऐं किन सुविधा केन्द्रों में किशोर/ किशोरियों के लिए उपलब्‍ध कराई जानी चाहिऐं? (खासतौर पर सामुदायिक स्‍तर, उप-केन्‍द्र, पीएचसी और सीएचसी के बारे में प्रोब करें)

1. Do you also provide products/treatment to the adolescents who come to your clinic? Probe about services provided to unmarried girls, and unmarried boys; Contraceptive counseling and supplies?? Referral?? Diagnosis and management of symptoms of genital infection?? Pregnancy care??

क्‍या आप भी किशोर/ किशोरियों को उत्‍पाद/ उपचार प्रदान करते हैं जो आपके क्‍लीनिक पर आते हैं? अविवाहित लड़कियों और अविवाहित लड़कों को प्रदान की गई सेवाओं के बारे में प्रोब करें; गर्भनिरोधक परामर्श और आपूर्तियां? रेफरल?? यौन संक्रमण के लक्षणों की पहचान और प्रबंधन?? गर्भावस्‍था देखभाल??

1. Can you tell me a recent instance in which you have provided contraceptives to an unmarried girl / an unmarried boy? Which method did she/he come for? Which method did you give her/him? What did you advice her/him? How do you decide which ones to be provided? How many adolescents have you provided contraceptive counseling in the last one week/month?

क्‍या आप मुझे हाल ही का कोई उदाहरण बता सकते हैं जिसमे आपने किसी अविवाहित लड़की/ किसी अविवाहित लड़के को गर्भनिरोधक प्रदान किये हों? वह कौन से तरीके के लिए आया था/ आई थी? आपने कैसे निर्णय लिया कि कौन सा तरीका प्रदान करना है? आपने पिछले एक हफ्‍ते/ महीने में कितने किशोर/ किशोरियों को गर्भनिरोधक परामर्श दिये हैं?

1. Can you tell me a recent instance in which you have provided counselling or advice in preventing unsafe abortions in a pregnant adolescent? What method did you advice her?

क्‍या आप मुझे हाल ही का कोई उदाहरण बता सकते हैं जिसमे आपने किसी गर्भवती किशोरी/ लड़की में असुरक्षित गर्भपात से बचाव के लिए परामर्श या सलाह दी? आपने उसे कौन से तरीके की सलाह दी?

1. Have you made any referrals? Can you tell me a recent instance in which you have referred an unmarried girl/ an unmarried boy to a health facility? What was the problem experienced by her/him? Where did you refer her/him?

क्‍या आपने कोई रेफरल्‍स किये हैं? क्‍या आप मुझे हाल ही का कोई उदाहरण बता सकते हैं जिसमे आपने किसी अविवाहित लड़की/ अविवाहित लड़के को किसी स्‍वास्‍थ्‍य सुविधा के लिए रेफर किया हो? उसे किन समस्‍याओं का अनुभव हो रहा था? आपने उसे कहां के लिए रेफर किया?

1. Are the services provided at this facility friendly to adolescents and is there a need for health providers to make some changes in their practice pattern to make the services morefriendly? What all additional services do you expect to be available at the Ujala clinic? What are your suggestions for improvement?

क्‍या इस सुविधा पर प्रदान की जाने वाली सेवाऐं किशोर/ किशोरियों के अनुकूल हैं और क्‍या सेवाओं को ज्‍यादा अनुकूल बनाने के लिए स्‍वास्‍थ्‍य प्रदाता को अपने अभ्‍यास तरीके मे कुछ बदलाव करने की जरूरत है? आप उजाला क्‍लीनिक्‍स में कौन सी अतिरिक्‍त सेवाओं के उपलब्‍ध होने की उम्‍मीद करते हैं? बेहतरी/ सुधार के लिए आपके क्‍या सुझाव हैं?

Thank you so much for giving so much time for us. Do you have any specific recommendations for improving the capacity of counsellors like you to better serve adolescents in your village? If yes, what? Do you have any suggestions for improving services at the clinic? If yes, what?

हमारे लिए इतना समय देने के लिए आपका बहुत-बहुत धन्‍यवाद। क्‍या आपके पास अपने गांव में किशोर/ किशोरियों को बेहतर सेवा देने के लिए आप जैसे काउंसलर्स की क्षमता बेहतर बनाने के लिए क्‍या कोई विशेष सिफारिशें/ सुझाव हैं? यदि हां, तो क्‍या? क्‍या क्‍लीनिक पर सेवाऐं बेहतर बनाने के लिए आपके पास कोई सुझाव हैं? यदि हां, तो क्‍या?
